# Novel Quantum Criticality in Two Dimensional Topological Phase transitions

**DOI:** 10.1038/srep19198

**Published:** 2016-01-21

**Authors:** Gil Young Cho, Eun-Gook Moon

**Affiliations:** 1 Department of Physics, Korea Advanced Institute of Science and Technology, Daejeon 305-701, Korea

## Abstract

Topological quantum phase transitions intrinsically intertwine self-similarity and topology of many-electron wave-functions, and divining them is one of the most significant ways to advance understanding in condensed matter physics. Our focus is to investigate an unconventional class of the transitions between insulators and Dirac semimetals whose description is beyond conventional pseudo relativistic Dirac Hamiltonian. At the transition without the long-range Coulomb interaction, the electronic energy dispersion along one direction behaves like a relativistic particle, linear in momentum, but along the other direction it behaves like a non-relativistic particle, quadratic in momentum. Various physical systems ranging from TiO_2_-VO_2_ heterostructure to organic material *α*-(BEDT-TTF)_2_I_3_ under pressure have been proposed to have such anisotropic dispersion relation. Here, we discover a novel quantum criticality at the phase transition by incorporating the 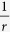 long range Coulomb interaction. Unique interplay between the Coulomb interaction and electronic critical modes enforces not only the anisotropic renormalization of the Coulomb interaction but also marginally modified electronic excitation. In connection with experiments, we investigate several striking effects in physical observables of our novel criticality.

Quantum criticality and topology are two of the main impetuses of modern condensed matter physics. Self-similarity of many-electron wave-functions associated with quantum criticality^1^,^2^,^3^ unveils emergent universality of physical observables, and topology of the electronic wave-functions manifests itself as various fascinating topological insulators and associated quantized responses^4^,^5^,^6^,^7^. The two striking characteristics of the wave-function are naturally and inevitably intertwined at topological quantum phase transitions.

Long-range 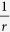 Coulomb interaction between electrons induces striking screening effects near the topological phase transitions. Electronic critical modes and the Coulomb interaction are intrinsically correlated, so non-trivial quantum criticality usually appears^8^,^9^,^10^,^11^,^12^,^13^. For example, quasi-particles lose their stability due to the Coulomb interaction and the ground state becomes quantum critical non-Fermi liquid with emergent full rotational symmetry in quadratic band touching semimetals, which is near three dimensional (3d) topological insulator^8^.

In two dimensions (2d), the Coulomb interaction becomes more special. It is because the Coulomb potential originally lives in 3d but electrons are confined in 2d. Thus the electrons in 2d feels the dimensionally different interaction, originating from 3d. Since correlation and fluctuation are enhanced in lower dimensions, one may expect stronger interplay between the Coulomb interaction and critical modes in a topological phase transition, and indeed we find the novel quantum criticality in a class of 2d topological quantum phase transitions.

Conventional 2d topological phase transitions between two topologically distinct insulators are described by the pseudo-relativistic Dirac fermion theory 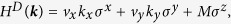 with Pauli matrices in band index spinor space. Here the topological nature of the transition is captured by the change in the Berry curvature of the wave-function depending on the sign of *M*, and different patterns of opening up band gaps at separate Dirac points represent different topological insulator phases when supplemented with proper symmetries. The long-range Coulomb interaction at the critical point 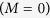 induces intriguing logarithmic modification of the Dirac velocities, so not only rotational symmetry at the critical point emerges but also important interaction effects appear whose structure has been extensively studied in literature^14^,^15^ in connection with charge-neutral mono-layer graphenes. We emphasize that the isotropic 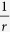 Coulomb interaction dominates microscopic anisotropy of electrons in this case.

Here we focus on a different class of the topological phase transitions whose electronic Hamiltonian is


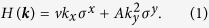


With a tuning parameter *m*, energy spectrum of 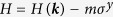 is 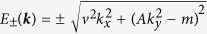. The two phases are determined by the sign of the tuning parameter. With a positve *A* and 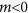, energy spectrum is gapped, so the ground state is an insulator. On the other hand, with 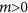, the zero-energy points appear at two points in momentum space, 
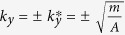
. By expanding the Hamiltonian (1) near these points, we obtain 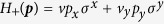 around the point 
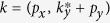
 with 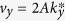. Thus the ground state is a 2d Dirac semimetal. Thus, it is clear that our model Hamiltonian describes a phase transition between a (either topological or trivial) insulator and a Dirac semimetal in 2d.

The Hamiltonian (1) has been suggested in various physical systems, ranging from TiO_2_-VO_2_ oxide heterostructures^16^,^17^,^18^ and the organic material *α*-(BEDT-TTF)_2_I_3_ under pressure^19^,^20^,^21^ to optical lattice systems^22^,^23^,^24^. For example, in the oxide heterostructure TiO_2_-VO_2_ layers^16^,^17^,^18^, there is a metal-insulator transition as the number of layers is changed. At the certain number of layers, the first-principle band structure calculation^16^,^17^,^18^ reveals that there should be the anisotropic semimetal (1). Furthermore, the structure of the Hamiltonian (1) is similar to that of the notorious quantum criticality problem with Fermi surfaces in 2d^25^,^26^,^27^,^28^,^29^,^30^ whose scaling of the dispersion along the radial direction to the Fermi surface is linear in momentum while that along the perpendicular direction is quadratic in momentum. Due to the similarity in dispersions, we expect that our analysis might shed some light on understanding the quantum criticality with Fermi surfaces despite of the finite density of states in the Fermi surface case.

In this work, we show, by using the systematic renormalization group (RG) method, that the long range interaction strongly changes the nature of the eigenstates of the non-interacting Hamiltonian. We find a novel quantum criticality characterized by *both* anisotropically renormalized and marginal Coulomb interactions which is in sharp contrast to other quantum criticalities. The anisotropic marginal quantum criticality is out of intricate interplay between the long range Coulomb interaction in 2d and the critical electron modes, and we emphasize its striking properties by calculating physical observables.

## Models with Coulomb Interaction

We start with the theory incorporating the electron Hamiltonian with the long-range 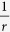 Coulomb interaction,


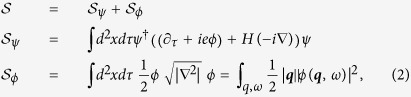


where 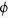 mediates Coulomb interaction between electrons 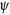. The short-hand writing 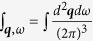 is used. Hereafter, all integrations are defined with the short-distance (or high-energy) ultra-violet (UV) cutoff. 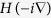 is the Fourier transformation of 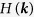 (1), and the bare gauge boson propagator 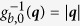 represents the long-range 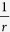 Coulomb interaction. For future convenience, we introduce a dimensionless coupling constant, the fine structure constant 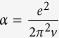, which measures the “strength” of Coulomb interaction.

**Figure 1:**
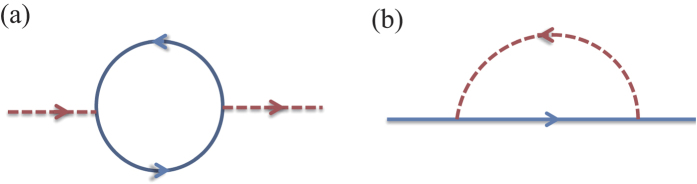
Diagrams for (a) the boson self-energy Π(*q*) and (b) the fermion self-energy 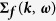. Here the dotted line represents the boson propagator 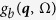 and the solid line represents the fermion propagator 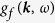.

We investigate the stability of the theory by the lowest order perturbation calculation, in particular, by calculating the bosonic self-energy whose Feynman diagram representation is in Fig. 1(a),


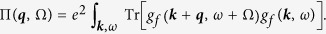



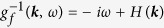
 is used. It is straightforward to evaluate the integral (see the supplementary information I for detail), and we find that


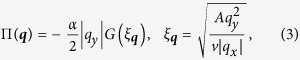


where 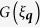 is the function of the *dimensionless parameter* 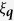. Hereafter, we drop the frequency dependence in the boson self-energy since we are only interested in the instantaneous Coulomb interaction. The full functional form of 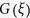 is not important. Thus we will not present it here and plot it only in the supplementary information I. Instead, the asymptotic behavior of 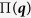 in each direction is extracted


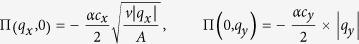


with the numeric constants 
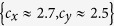
. Notice that the boson self-energy is independent of the UV cutoff which signal a novel quantum criticality in our system.

It is clear that the perturbation becomes more important than the original bare term along 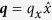,


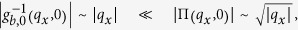


in the limit 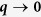. Thus we conclude that the action (2) is *unstable* under the fermion-gauge boson coupling.

The instability from the perturbative calculation often indicates the presence of the stable strong-coupling fixed point which can be accessed by large-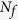 analysis with the number of fermion flavors 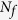. The large-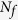 analysis starts with adding the bosonic self-energy to the boson bare term,


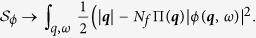


The schematic representation of the inverse of the corrected boson propagator 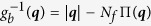 is


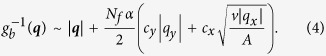


The limit 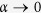 recovers the unstable bare action (2), and we investigate the opposite limit 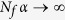 where we drop the bare term 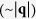.

Using this corrected boson propagator, we calculate the fermion self-energy in Fig. 1(b)


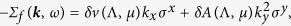


obtained by expanding the self-energy near 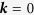 with the UV and IR cutoffs, 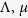. Straightforward calculation gives


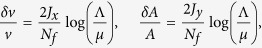


with the two dimensionless constants, 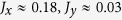 (see supplementary information III for detail). We notice that the instantaneous nature of the Coulomb propagator enforces no vertex correction through the Ward identity.

Therefore, the RG flow equations, i.e., beta functions, for *v* and *α* can be derived by changing the ratio, 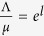,


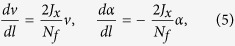


near the strong-coupling fixed point. It is clear from the RG equations that the fine structure constant *α* decreases with the anomalous dimension of the velocity, 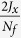. This concludes that the strong-coupling fixed point is *unstable*.

Our controlled analysis near the two extreme limits (standard perturbation and large-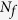 analysis) clearly shows that both the fixed points are unstable. Then it is obvious that the stable fixed point should be in the intermediate regime, which is difficult to access in a fully controlled way. Thus, we study the fixed point with the standard momentum-shell RG and check *a posteriori* its validity by self-consistency.

In the momentum-shell RG analysis, we remark that the non-analytic dependence 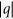 of the Coulomb interaction does not receive correction from integrating out higher-momentum modes. Thus, we first keep the seemingly irrelevant 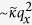 term in the boson action,


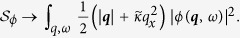


It turns out that the following three dimensionless parameters determine the RG flows


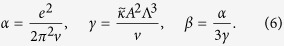


Evaluating Feynman diagrams in Fig. 1 gives the renormalized action 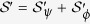. Here, we use the cutoff scheme such that we integrate along the 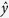-directional momentum 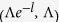 with 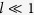 after integrating out the 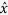-direction momentum and frequency. On integrating out the higher-momentum modes, three parameters are renormalized as


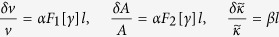


We find that the functions 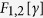 are non-negative near 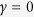 whose specific forms are illustrated in supplementary information IV. The RG flow equations of 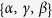 are


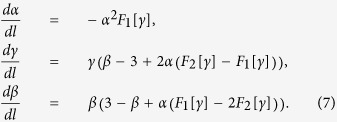


The two fixed points are, 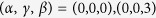, and it is easy to show that the former is unstable and the latter is stable. At the stable fixed point 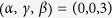, the boson propagator receives a large anomalous scaling dimension, which can be understood as 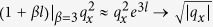 (because 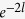 is the scaling factor of 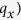. Such large anomalous dimension indicates that the momentum-shell RG is not controlled and *a priori* not reliable.

At the stable fixed point, the effective bosonic action becomes


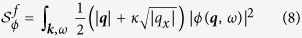


which is very similar to the large-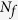 calculation with one important difference; a new coupling constant 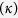 with UV cutoff scale naturally enters in contrast to the large-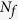 calculation where the coefficient of 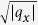 is 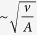 (see equation (4)) which depends on the other parameters 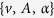. Here the new dimensionful parameter 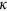 appears in the bosonic part at the intermediate coupling regime.

With this intuition in hand, we investigate the stability of the new fixed point by taking equation (8) as the bare boson action and performing the momentum-shell RG near this fixed point. Remarkably, we find that the velocity *v* and inverse mass *A* receives the same corrections at the fixed point


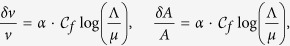


The same correction is another evidence for our fixed point to be stable since the ratio 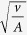 appearing in the boson self-energy 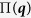 becomes constant. Notice that the remarkable same correction also appears in Fermi surface quantum criticalities with very different physical reasons which also supports stability of our fixed point^26^. It is manifest that the gapless excitation structure of our system is completely different from that of Fermi surfaces (lines) in 2d. Only nodal point excitation appears in our system. However, the low energy scaling structures of the two systems are same considering the patch theory of Fermi surfaces in 2d ; one momentum direction has linear scaling while the other one has quadratic scaling. We believe this unexpected similarity is the source of the similar behaviors in the beta functions. It would be very intriguing to find more similarity and difference of two systems’ quantum criticalities, which we leave for future work.

The beta functions around the novel fixed point are


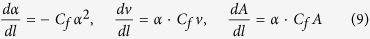


where 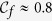 calculated in the supplementary information V.

We remark that the hard momentum cutoff scheme is only used for simplicity and illustration. It is shown that our results are independent of cutoff schemes in the supplementary information VIII.

The above RG flow structure (9) is unique to this fixed point. The beta functions contain the fine structure constant *α* in contrast to those of the large-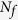 calculation (5) in which *α* is absent, and here both 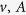 receive the *same* logarithmic corrections, which are proportional to *α*. Thus, the fine structure constant *α* decreases and the fermion only receives the logarithmic corrections, which indicates the fixed point is *stable*. Naturally, as in mono-layer graphene, marginal Fermi liquid behaviors are expected with higher order corrections^31^.

**Figure 2:**
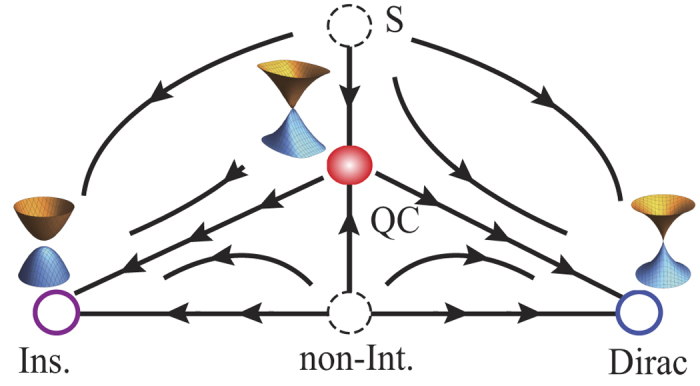
Proposed RG flow. The horizontal axis is for the tuning parameter *m* of the quantum criticality equation (1) and the vertical axis is for the strength of Coulomb interaction. There are two stable fixed points, insulators (‘Ins’) and Dirac semimetal (‘Dirac’). The two unstable critical points are illustrated with dashed circles, non-interacting (‘Non-Int.’) and strong-coupling fixed point (‘S’). And the stable critical point is the filled circle (‘QC’). The critical point is characterized by the definite anisotropic scaling and the logarithmic corrections to mass and velocity. Near the fixed points (Ins, Dirac, QC), one-particle spectrum with the Coulomb interaction is illustrated.

Based on the calculations and intuitions, a schematic RG flow can be deduced as in Fig. 2 which summarizes our main results. Our controlled calculation shows the non-interacting critical point (Non-Int.) and the strong coupling fixed point (S) are unstable and the RG flow comes out of the both points and flow into the intermediate fixed point (QC), which is characterized by the definite anisotropic scaling of bosons and electrons and the single logarithmic corrections to velocity and inverse mass.

## Experimental Signatures

We now investigate the physical consequences of both the anisotropy and marginal irrelevance of the renormalized Coulomb potential at the novel intermediate critical point.

First of all, with the beta functions of *v* and *A* (9), we can find logarithmic corrections to all physical quantities. The parameters 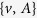 at the temperature scale *T* are


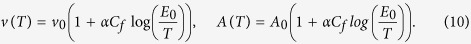


Here 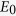 is the bandwidth or the UV cutoff of the theory (1). 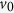 and 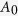 are the bare parameters at the highest energy scale 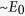. The logarithmic corrections in 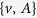 may be observed in quasi-particle experiments as in graphene, for example, angle resolved photo-emission spectroscopy (ARPES)^32^ or quantum oscillation.

Furthermore, thermodynamic quantities such as specific heat and compressibility also show logarithmic corrections. Specific heat and compressibility of the unstable free electron fixed point are 
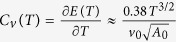
 and 
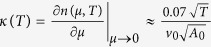
 in which 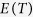 is the (thermal-averaged) energy density per volume as the function of temperature *T* and 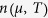 is the density of the electron per volume as the function of chemical potential *μ* and temperature *T*. But at the novel fixed point, the logarithmic corrections give


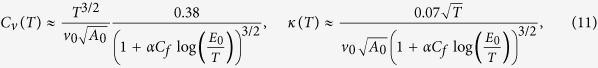


at the temperature *T* by following the reference^14^.

Secondly, we can see the effect of the anisotropic renormalization of the gauge boson via the screening charge when a single impurity charge *Z* is introduced at 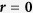. At the level of the linear response theory, the screening charge is 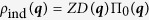, in which 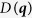 is the propagator of the gauge boson. We are interested in the *directional* behaviors of the screening charge and hence define the integrated screening charges 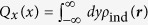 and 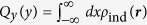 along 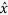 and 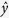. Here we will contrast the extremely different behaviors of the screening charges between the free fixed point and the non-trivial fixed point.

At the free fixed point, we ignore the corrections to the gauge boson propagator and use 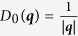. Following the straightforward calculation in supplementary information VI, we find 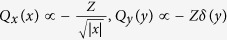 whose sign is the opposite of the impurity charge *Z*.

On the other hand, at the non-trivial anisotropic fixed point in which we use the renormalized boson propagator 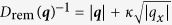, we find the asymptotic behaviors of the screening charges


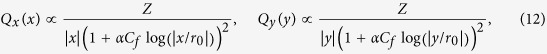


where 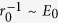 is the UV cutoff. Here the sign of the screening charge is the same as the impurity charge *Z*, which is reminiscent of graphene case^33^.

From the above calculations, we see that the asymptotic scaling behaviors of the screening charges in distance from the impurity along 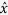 and 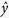 are surprisingly isotropic. The isotropic scaling in both the directions is originated from the facts that the scaling of 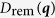 is identical to that of 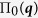 and that 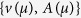 at the energy scale *μ* receive the same logarithmic corrections as (9). Hence this isotropic scaling behaviors are truly from the effects of interactions between the electrons and the gauge boson.

## Discussion and Conclusion

The presence of the novel fixed point implies that the electrons and gauge bosons are strongly correlated. At low energy, electrons and gauge bosons affect each other, so the Coulomb interaction mediated by the bosons becomes anisotropic and electrons receive back-reaction from the renormalized anisotropic Coulomb interaction. Thus, the Coulomb interaction behaves differently from that of most critical systems where it enforces low-energy isotropy of electronic modes^8^,^14^,^31^,^34^. Also, notice that the ground state of our fixed point has marginally well-defined quasi-particles as those in graphene, which is in contrast to non-Fermi liquids with non-zero anomalous dimensions. In Table 1, the comparison with other quantum criticality associated with topological phase transitions is summarized.

Our novel quantum criticality can be experimentally tested in the systems such as VO_2_-TiO_2_ heterostructure. Near the critical point, optical conductivity shows anisotropy inherited from the electron band structure. Straightforward calculation with current operators 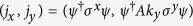 gives


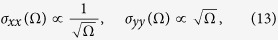


upto logarithmic corrections from the Coulomb interaction (see the supplementary information VII). However, as shown in the previous section, the screening charge due to the charged impurity, which can be measured in principle by scanning tunneling microscopy (STM), shows qualitatively isotropic behaviors. Such discrepancy between the two experiments is a smoking gun of the novel quantum criticality in addition to thermodynamics quantities such as specific heat.

It is worth to mention that disorder scattering in the non-interacting electrons (1) is relevant^35^, so our results work better for cleaner samples. We expect that there will be an intriguing interplay between the anisotropic Coulomb interaction and impurity scattering at the novel critical point, which we leave for the future problem.

In conclusion, we have investigated the quantum criticality of the anisotropic semimetal which can be thought as the critical point between topological insulators and Dirac semimetal in two spatial dimensions. At the low-energy limit, we found the novel fixed point out of the interplay between critical electron modes and the long-range 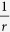 Coulomb interaction. The non-trivial anisotropic renormalization of the Coulomb interaction and the logarithmic corrections manifest at various physical quantities including screening charge when the impurity charge is introduced. Surprisingly we have shown that the scaling behavior of the screening charge in distance from the impurity is isotropic despite of the underlying anisotropic nature of the system.

Note added : After the completion of the paper, we became aware of the independent work by H. Isobe, B.-J. Yang, A. Chubukov, J. Schmalian, and N. Nagaosa [ref. (36)]. Similarity and differences between our work and theirs are discussed in supplementary information.

## Additional Information

**How to cite this article**: Cho, G. Y. and Moon, E.-G. Novel Quantum Criticality in Two Dimensional Topological Phase transitions. *Sci. Rep.* **6**, 19198; doi: 10.1038/srep19198 (2016).

## Supplementary Material

Supplementary Information

## Figures and Tables

**Table 1:** Comparison with the quantum criticalities in various semimetallic systems.

Systems	Excitation	Coulomb
2D Dirac^14^,^31^	marginal q.p.	iso., marginally irr.
3D Dirac^34^	marginal q.p.	iso., marginally irr.
3D Quadratic^8^	no q.p.	iso., relevant
3D Anisotropic^10^	q.p.	aniso., irr.
2D Anisotropic	marginal q.p.	aniso., marginal

Here the second column represents types of allowed excitation. “q.p.” is for quasi-particle. The third column represents characteristics of screened Coulomb interaction. “iso.” is for isotropic, “aniso.“ is for anisotropic, and “irr.” is for irrelevant.
