# Supplementary material for: Novel Quantum Criticality in Two Dimensional Topological Phase transitions
Source: Sci Rep. 2016 Jan 21;6:19198. doi: 10.1038/srep19198 (PMC4726365; doi:10.1038/srep19198)
Supplement: Supplementary Information [file srep19198-s1.pdf]

Supplementary Information for “Novel Quantum Criticality in Two Dimensional Topological Phase transitions”

Gil Young Cho and Eun-Gook Moon
Department of Physics, Korea Advanced Institute of Science and Technology, Daejeon 305-701, Korea
(Dated: December 16, 2015)

I. POLARIZATION BUBBLE

In this supplemental method, we present the detailed calculation of the polarization, or the boson self-energy $\Pi(\boldsymbol{k})$ appearing in the main text. For convenience, we assume N-copies of the electrons coupled to the gauge boson. If we are interested in the only one copy, i.e., physical limit, one can simply take $N = 1$ at the end of the calculation.

A. Deivation of Polarization

In the leading order in $1/N$ expansion, the boson self-energy is simply the two-leg one-loop diagram. The fermion propagator appearing in the loop diagram is

$$g_f(\boldsymbol{k},\omega) = \frac{1}{-i\omega + H(\boldsymbol{k})}, \tag{1}$$

where $H(\boldsymbol{k})$ is the free fermion Hamiltonian equation (6) in the main text. It is convenient to write the propagator in the following form

$$\begin{aligned} g_f(\boldsymbol{k},\omega) &= \frac{1}{-i\omega + H(\boldsymbol{k})}, \\ &= \sum_{s=\pm} \frac{1}{-i\omega + E_s(\boldsymbol{k})} P_s(\boldsymbol{k}), \end{aligned} \tag{2}$$

with the energy $E_s(\boldsymbol{k}) = sE(\boldsymbol{k}) = s\sqrt{v^2k_x^2 + A^2k_y^4}$ and the projection $P_s(\boldsymbol{k})$

$$P_s(\boldsymbol{k}) = \frac{1}{2}\Big(1 + s\frac{H(\boldsymbol{k})}{E(\boldsymbol{k})}\Big). \tag{3}$$

Now the polarization is given by

$$\Pi(\boldsymbol{p},\Omega) = \frac{e^2}{N}\int_{\boldsymbol{k},\omega} \mathrm{Tr}\Big[g_f(\boldsymbol{k}+\frac{\boldsymbol{p}}{2},\omega+\Omega)g_f(\boldsymbol{k}-\frac{\boldsymbol{p}}{2},\omega)\Big]. \tag{4}$$

Performing the contour integral over ω and the trace $\mathrm{Tr}[\cdot]$, we finally have

$$\Pi(\boldsymbol{p},\omega) = -e^2N\int_{\boldsymbol{k}} \frac{\mathcal{P}(\boldsymbol{k},\boldsymbol{p})\big(E(\boldsymbol{k}+\frac{\boldsymbol{p}}{2}) + E(\boldsymbol{k}-\frac{\boldsymbol{p}}{2})\big)}{\omega^2 + \big(E(\boldsymbol{k}+\frac{\boldsymbol{p}}{2}) + E(\boldsymbol{k}-\frac{\boldsymbol{p}}{2})\big)^2}, \tag{5}$$

in which $\mathcal{P}(\boldsymbol{k},\boldsymbol{p})$ is the projection operator

$$\mathcal{P}(\boldsymbol{k},\boldsymbol{p}) = 1 - \frac{\sum_{\mu=1,2}\epsilon_\mu(\boldsymbol{k}+\frac{\boldsymbol{p}}{2})\epsilon_\mu(\boldsymbol{k}-\frac{\boldsymbol{p}}{2})}{E(\boldsymbol{k}+\frac{\boldsymbol{p}}{2})E(\boldsymbol{k}-\frac{\boldsymbol{p}}{2})}, \tag{6}$$

where $\epsilon_1(\boldsymbol{k}) = vk_x$ and $\epsilon_2(\boldsymbol{k}) = Ak_y^2$ are the appropriate functions.

B. Calculation of Polarization

To evaluate the function $\Pi(\boldsymbol{k}) \equiv \Pi(\boldsymbol{k},\omega=0)$ in equation(5), we use the change of variables as following:

$$k_x = p_x x, \quad \text{and} \quad k_y = \sqrt{\frac{v|p_x|}{A}}y, \tag{7}$$

which gives rise to the following expression.

$$\Pi(\boldsymbol{p}) = -\frac{e^2N}{4\pi^2 v}\sqrt{\frac{v|p_x|}{A}}\int_{-\infty}^{\infty}dx\int_{-\infty}^{\infty}dy\ f_\xi(x,y), \tag{8}$$

where $\xi = |p_y|\sqrt{\frac{v|p_x|}{A}}$ and$f_\xi(x,y)$ is the function

$$f_\xi(x,y) = \frac{\Big[1 - \frac{x^2-\frac{1}{4}+(y^2-\frac{\xi^2}{4})^2}{\sqrt{(x+\frac{1}{2})^2+(y+\frac{\xi}{2})^4}\sqrt{(x-\frac{1}{2})^2+(y-\frac{\xi}{2})^4}}\Big]}{\sqrt{(x+\frac{1}{2})^2+(y+\frac{\xi}{2})^4}+\sqrt{(x-\frac{1}{2})^2+(y-\frac{\xi}{2})^4}}. \tag{9}$$

We introduce a function

$$F(\xi) = \int_{-\infty}^{\infty}dx\int_{-\infty}^{\infty}dy\ f_\xi(x,y). \tag{10}$$

Then it is easy to see $F(\xi) = F(-\xi)$, i.e., $F(\xi) = F(|\xi|)$. Now, we can see that

$$F(|\xi|\to 0) \to c_x, \quad \text{and} \quad F(|\xi|\to\infty) \to c_y\xi, \tag{11}$$

where $c_x \approx 2.7$ and $c_y \approx 2.5$. We plot $F(\xi)$ in the Fig. 1. From the numerical calculation, we can extract both the constants. With these in hand, we can write the polarization as

$$\begin{aligned} \Pi(\boldsymbol{p}) &= -\frac{e^2N}{4\pi^2 v}|p_y|\frac{F(|\xi|)}{|\xi|} = -\frac{e^2N}{4\pi^2 v}|p_y|G(|\xi|) \\ &= -\frac{\alpha N}{2}|p_y|G(|\xi|), \end{aligned} \tag{12}$$

where we have used $G(|\xi|) = \frac{F(|\xi|)}{|\xi|}$ and $\alpha = \frac{e^2}{2\pi^2 v}$. From equation(11), we can see that

$$G(|\xi|\to 0) \to \frac{c_x}{\xi}, \quad \text{and} \quad G(|\xi|\to\infty) \to c_y, \tag{13}$$

and $G(|\xi|) > 0$ for any ξ.

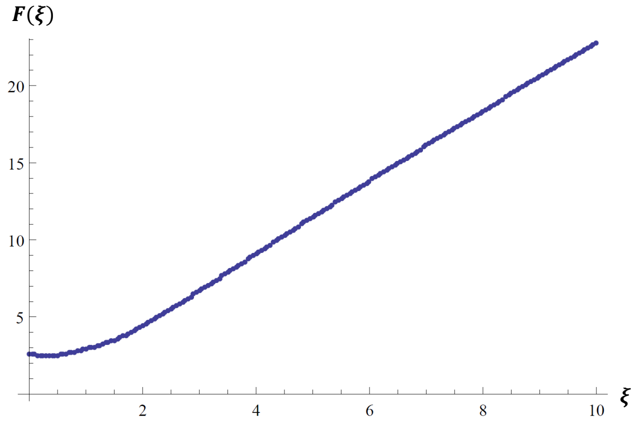

FIG. 1. Plot of $F(\xi)$

Systems	Bare	Boson Self-energy
2D Dirac[1,2]	$\lvert\boldsymbol{q}\rvert$	$\lvert\boldsymbol{q}\rvert$
3D Dirac[3]	$\boldsymbol{q}^2$	$\boldsymbol{q}^2 \log\Lambda$
3D Quadratic[4]	$\boldsymbol{q}^2$	$\lvert\boldsymbol{q}\rvert$
3D Double Weyl[5,6]	$\boldsymbol{q}^2$	$q_\perp^2 \log\Lambda + \lvert q_z\rvert$
3D Anisotropic[7]	$\boldsymbol{q}^2$	$\Lambda \lvert q_\perp\rvert^2 + \lvert q_z\rvert^{\frac{3}{2}}$
2D Anisotropic	$\lvert\boldsymbol{q}\rvert$	$\sqrt{\lvert q_x\rvert} + \lvert q_y\rvert$

TABLE I. Bare and self-energy of boson propagators in various quantum criticalities. The second and thir column represent the bare and the one-loop self-energy of various quantum criticalities. Λ is the UV cutoff and only schematic behaviors are shown. Note that 3D quantum criticalities depend on the UV cutoff except the non-Fermi liquid quadratic band touching.

II. SCREENING EFFECTS IN QUANTUM CRITICALITIES

Here, we summarize bare and self-energy of Coulomb boson propagators in various quantum criticalities. The one-loop boson self-energy is obtained in the standard way. With different electron Hamiltonian for each case, we obtain the self-energy which is illustrated in Table I.

III. FERMION SELF-ENERGY AND RG EQUATIONS IN STRONG-COUPLING LIMIT

Here we compute the fermion self-energy at the large-N limit to extract the RG equations. We start with the expression for the fermion self-energy with the corrected boson propagator

$$\Sigma_f(\boldsymbol{k},\omega) = -\frac{e^2}{2}\int_{\boldsymbol{q}} \frac{H(\boldsymbol{q}+\boldsymbol{k})}{E(\boldsymbol{q}+\boldsymbol{k})} \frac{1}{|\boldsymbol{q}| + \frac{\alpha N}{2}|q_y| G(\xi_{\boldsymbol{q}})}. \quad (14)$$

To extract out the correction to the bare parameters $\{v, A\}$, we expand the $\Sigma_f(\boldsymbol{k},\omega)$ in $\boldsymbol{k}$ near $\boldsymbol{k}=0$,

$$-\Sigma_f(\boldsymbol{k}) = v k_x \sigma^x \tilde{I}_x + A k_y^2 \sigma^y \tilde{I}_y. \quad (15)$$

Here $\{\tilde{I}_x, \tilde{I}_y\}$ are the integrals

$$\tilde{I}_x = \frac{e^2}{8\pi^2}\int dq_x dq_y \frac{A^2 q_y^4}{[v^2 q_x^2 + A^2 q_y^4]^{3/2}} \frac{1}{|\boldsymbol{q}| + \frac{\alpha N}{2}|q_y| G(\xi_{\boldsymbol{q}})},$$
$$\tilde{I}_y = \frac{e^2}{8\pi^2}\int dq_x dq_y \frac{v^4 q_x^4 - 5 v^2 q_x^2 A^2 q_y^4}{[v^2 q_x^2 + A^2 q_y^4]^{5/2}} \frac{1}{|\boldsymbol{q}| + \frac{\alpha N}{2}|q_y| G(\xi_{\boldsymbol{q}})}. \quad (16)$$

Apparently, the integrals are divergent and needed to be regulated. We regulate the integrals by

$$\int dq_x dq_y \to \Big(\int_\mu^\Lambda dq_x + \int_{-\Lambda}^{-\mu} dq_x\Big)\int_{-\infty}^{\infty} dq_y, \quad (17)$$

where the UV and IR cutoffs $\{\Lambda, \mu\}$ are introduced.

With the expression equation(15), we find

$$\delta v = -\frac{1}{\mathrm{Tr}[\sigma^0]}\mathrm{Tr}\Big[\sigma^x \frac{\delta\Sigma_f(\boldsymbol{k},\omega)}{\delta(k_x)}\Big]_{\boldsymbol{k}\to\boldsymbol{0},\omega\to 0}$$
$$= v\tilde{I}_x$$
$$\delta A = -\frac{1}{\mathrm{Tr}[\sigma^0]}\mathrm{Tr}\Big[\sigma^y \frac{\delta\Sigma_f(\boldsymbol{k},\omega)}{\delta(k_y^2)}\Big]_{\boldsymbol{k}\to\boldsymbol{0},\omega\to 0}$$
$$= A\tilde{I}_y \quad (18)$$

A. Strong-coupling limit

We first evaluate the integrals equation(16) when $\alpha \to \infty$.

$$\tilde{I}_x = \frac{e^2}{4\pi^2\alpha N}\int dq_x \int dq_y \frac{A^2 q_y^4}{[v^2 q_x^2 + A^2 q_y^4]^{3/2}} \frac{1}{|q_y| G(\xi_{\boldsymbol{q}})},$$
$$\tilde{I}_y = \frac{e^2}{4\pi^2\alpha N}\int dq_x \int dq_y \frac{v^4 q_x^4 - 5 v^2 q_x^2 A^2 q_y^4}{[v^2 q_x^2 + A^2 q_y^4]^{5/2}} \frac{1}{|q_y| G(\xi_{\boldsymbol{q}})}, \quad (19)$$

in which the cutoff scheme equation(17) is assumed. We demonstrate the detail of evaluations only for $\tilde{I}_x$.

$$\tilde{I}_x = \frac{e^2}{4\pi^2\alpha N}\int dq_x \int dq_y \frac{A^2 q_y^4}{[v^2 q_x^2 + A^2 q_y^4]^{3/2}} \frac{1}{|q_y| G(\xi_{\boldsymbol{q}})},$$
$$= \frac{e^2}{4\pi^2\alpha N v}\int \frac{dq_x}{q_x}\int \frac{dq_y}{|q_y|} \frac{\frac{A^2 q_y^4}{v^2 q_x^2}}{[1 + \frac{A^2 q_y^4}{v^2 q_x^2}]^{3/2}} \frac{1}{G(\xi_{\boldsymbol{q}})}$$
$$= \frac{1}{2N}\int \frac{dq_x}{q_x}\int_{-\infty}^{\infty} d\xi \frac{\xi^3}{(1+\xi^4)^{3/2}} \frac{1}{G(\xi)}$$
$$= \frac{2}{N}\log(\frac{\Lambda}{\mu})\int_0^{\infty} d\xi \frac{\xi^3}{(1+\xi^4)^{3/2}} \frac{1}{G(\xi)}$$
$$= \frac{2J_x}{N}\log(\frac{\Lambda}{\mu}) \quad (20)$$

where

$$J_x = \int_0^\infty d\xi \frac{\xi^3}{(1+\xi^4)^{3/2}} \frac{1}{G(\xi)}. \tag{21}$$

The integral is well-defined in that it has no divergence in the integral because

$$\begin{aligned} \frac{\xi^3}{(1+\xi^4)^{3/2}} \frac{1}{G(\xi)}\Big|_{\xi\to\infty} &\to \frac{1}{\xi^3}\frac{1}{c_x} \propto \frac{1}{\xi^3} \\ \frac{\xi^3}{(1+\xi^4)^{3/2}} \frac{1}{G(\xi)}\Big|_{\xi\to 0} &\to \xi^3 \frac{1}{c_y/\xi} \propto \xi^4 \end{aligned} \tag{22}$$

The integral can be evaluated numerically by using the numeric value of $G(\xi)$, and we have obtained $J_x \approx 0.18$. The numeric value is not important but it is important to remember $J_x > 0$ because of $G(|\xi|) > 0$.

Similarly, we can write $\tilde{I}_y$ as following.

$$\tilde{I}_y = \frac{2J_y}{N} \log(\frac{\Lambda}{\mu}), \tag{23}$$

where J_y is the definite integral

$$J_y = \int_0^\infty \frac{d\xi}{\xi} \frac{1-5\xi^4}{(1+\xi^4)^{5/2}} \frac{1}{G(\xi)}. \tag{24}$$

The integeral is well-defined because

$$\begin{aligned} \frac{1-5\xi^4}{(1+\xi^4)^{5/2}} \frac{1}{\xi G(\xi)}\Big|_{\xi\to\infty} &\to \frac{-5}{\xi^7}\frac{1}{c_y} \propto \frac{1}{\xi^7} \\ \frac{1-5\xi^4}{(1+\xi^4)^{5/2}} \frac{1}{\xi G(\xi)}\Big|_{\xi\to 0} &\to \frac{1}{\xi}\frac{1}{c_x/\xi} \propto \frac{1}{c_x}. \end{aligned} \tag{25}$$

Hence there is no more IR divergence than $\log(\Lambda/\mu)$ appearing in equation(23) in the renormalization of A in the strong-coupling limit. The numeric value of J_y is obtained as 0.03.

Hence, under the RG step, we find that the velocity v and the inverse mass A are renormalized as follows.

$$\begin{aligned} v &\to v + \delta v = v(1 + \frac{2J_x}{N}\log(\Lambda)), \\ A &\to A + \delta A = A(1 + \frac{2J_y}{N}\log(\Lambda)), \end{aligned} \tag{26}$$

from which we deduce the RG flow equations,

$$\begin{aligned} \frac{dv}{dl} &= \frac{2J_x}{N} v, \\ \frac{dA}{dl} &= \frac{2J_y}{N} A, \end{aligned} \tag{27}$$

which have been shown in the main text.

Notice that the frequency dependence is trivial since only the instantaneous Coulomb propagator is used. If one incorporates frequency dependence, only the numeric numbers J_x, J_y are modified but not the structure of our calculation. The absence of the frequency dependence indicates no vertex correction by the Ward identity.

B. Finite Coupling Constant

Here we will discuss the fate of the finite coupling constant α which is now slightly away from the strong-coupling limit. In the prsence of the finite coupling constant α, the bare term $\sim |\boldsymbol{q}|$ in the boson propagator

$$g_b(\boldsymbol{q}, \omega) = \frac{1}{|\boldsymbol{q}| - \Pi(\boldsymbol{q})} \tag{28}$$

cannot be ignored. Hence the above calculations of $\alpha \to \infty$ should be properly changed to see the scaling behaviors of the parameters $\{v, A\}$.

Performing the straightforward calculation as the above, we find

$$\frac{\delta v}{v} = \alpha K_x(\Lambda, \mu), \quad \frac{\delta A}{A} = \alpha K_y(\Lambda, \mu) \tag{29}$$

The integrals $\{K_x, K_y\}$ are

$$\begin{aligned} K_x &= \int_\mu^\Lambda \frac{dq_x}{q_x} \int_0^\infty d\xi \frac{\frac{\xi^4}{(1+\xi^4)^{3/2}}}{(\xi^2 + \bar{A}(q_x))^{1/2} + \frac{\alpha N}{2}\xi G(\xi)}, \\ K_y &= \int_\mu^\Lambda \frac{dq_x}{q_x} \int_0^\infty d\xi \frac{\frac{1-5\xi^4}{(1+\xi^4)^{5/2}}}{(\xi^2 + \bar{A}(q_x))^{1/2} + \frac{\alpha N}{2}\xi G(\xi)}, \end{aligned} \tag{30}$$

where $\bar{A}(q_x) = \frac{Aq_x}{v}$ is the dimensionless number depending on the momentum q_x.

Before marching further into the details, we first make an assumption, $\bar{A}(q_x) < \bar{A}(\Lambda) \ll 1$. The condition $\bar{A}(\Lambda) \ll 1$ should be interpreted as following. First of all, $v\Lambda$ can be roughly thought as the bandwidth where the low-energy Hamiltonian equation (6) in the main text is the qualitatively correct description of the system. Note that A determines the curvature along k_y and v determines the velocity along k_x. Thus $\bar{A}(\Lambda) \ll 1$ means that the velocity along k_y is always much smaller than the velocity along k_x within the energy $E \le v\Lambda$, i.e., the dispersion along the k_y direction is always much flatter than that along the k_x direction. In other words, the spectrum is *extremely anisotropic* in that the equal energy contour is extended along k_y and shrinked along k_x.

At this stage, the assumption $\bar{A}(\Lambda) \ll 1$ looks like an ad-hoc assumption to evaluate the integrals equation(30). However, employeeing the momentum-shell RG approach at the weak-coupling limit $\alpha \to 0$ (as present in the main text and the next supplemental method), we can show $\bar{A} \ll 1$ at the fixed point.

With this condition in hand, we proceed to evaluate the integrals equation(30) approximately by taking $\bar{A} \to 0$,

$$\begin{aligned} K_x &= \log(\frac{\Lambda}{\mu})\tilde{J}_x(\alpha) \\ K_y &= \log(\frac{\Lambda}{\mu})\tilde{J}_y(\alpha) \end{aligned} \tag{31}$$

where $\tilde{J}_x > 0$ and $\tilde{J}_y$ are the constants depending on α whose detailed calculation can be found below. From

these, we find

$$\frac{\delta v}{v} = \alpha \log(\frac{\Lambda}{\mu})\tilde{J}_x(\alpha),$$
$$\frac{\delta A}{A} = \alpha \log(\frac{\Lambda}{\mu})\tilde{J}_y(\alpha). \quad (32)$$

Hence we find that

$$\frac{\delta \log(v)}{\delta \log(\Lambda)} = \alpha \tilde{J}_x(\alpha)$$
$$\frac{\delta \log(A)}{\delta \log(\Lambda)} = \alpha \tilde{J}_y(\alpha). \quad (33)$$

Thus the parameters $\{v, A\}$ in the bare fermion Hamiltonian equation (6) in the main text receive the logarithmic corrections, and the coupling constant α decreases along the renormalization group process.

1. Calculation of $\tilde{J}_x$ and $\tilde{J}_y$

Below we only present the detail for evaluating $\tilde{J}_x$ but it is straightforward to generalize to $\tilde{J}_y$.

$$\tilde{J}_x = \frac{e^2}{8\pi^2}\int dq_x dq_y \frac{A^2 q_y^4}{[v^2q_x^2 + A^2 q_y^4]^{3/2}} \frac{1}{|\boldsymbol{q}| + \frac{\alpha N}{2}|q_y|G(\xi_{\boldsymbol{q}})},$$
$$= \frac{e^2}{4\pi^2 v}\int_\mu^\Lambda \frac{dq_x}{q_x}\int_{-\infty}^{\infty} dq_y \frac{\frac{A^2q_y^4}{v^2q_x^2}}{(1+\frac{A^2q_y^4}{v^2q_x^2})^{3/2}} \frac{1}{|\boldsymbol{q}| + \frac{\alpha N}{2}|q_y|G(\xi_{\boldsymbol{q}})}$$
$$= \frac{\alpha}{2}\int_\mu^\Lambda \frac{dq_x}{q_x}\int_{-\infty}^{\infty} \frac{dq_y}{|q_y|} \frac{\xi^4}{(1+\xi^4)^{3/2}} \frac{1}{\sqrt{1+\frac{q_x^2}{q_y^2}} + \frac{\alpha N}{2}G(\xi)}$$
$$= \alpha\int_\mu^\Lambda \frac{dq_x}{q_x}\int_0^\infty \frac{d\xi}{\xi} \frac{\xi^4}{(1+\xi^4)^{3/2}} \frac{1}{\sqrt{1+\frac{\bar{A}(q_x)}{\xi^2}} + \frac{\alpha N}{2}G(\xi)}, \quad (34)$$

in which $\bar{A}(q_x) = \frac{A}{v}q_x$ and $q_y = \sqrt{\frac{vq_x}{A}}\xi$. We continue to evaluate this

$$\tilde{J}_x = \alpha\int_\mu^\Lambda \frac{dq_x}{q_x}\int_0^\infty \frac{d\xi}{\xi} \frac{\xi^4}{(1+\xi^4)^{3/2}} \frac{1}{\sqrt{1+\frac{\bar{A}(q_x)}{\xi^2}} + \frac{\alpha N}{2}G(\xi)}$$
$$= \alpha\int_\mu^\Lambda \frac{dq_x}{q_x}\int_0^\infty d\xi \frac{\xi^4}{(1+\xi^4)^{3/2}} \frac{1}{\sqrt{\xi^2+\bar{A}(q_x)} + \frac{\alpha N}{2}\xi G(\xi)} \quad (35)$$

Now assuming $\bar{A}(q_x) < \bar{A}(\Lambda) \ll 1$ which we will justify in the low-energy limit later, we can ignore the dependence on $\bar{A}$ in the integral to obtain

$$\tilde{J}_x \approx \alpha\int_\mu^\Lambda \frac{dq_x}{q_x}\int_0^\infty d\xi \frac{\xi^4}{(1+\xi^4)^{3/2}} \frac{1}{\xi + \frac{\alpha N}{2}\xi G(\xi)}$$
$$= \alpha\int_\mu^\Lambda \frac{dq_x}{q_x}\int_0^\infty d\xi \frac{\xi^3}{(1+\xi^4)^{3/2}} \frac{1}{1 + \frac{\alpha N}{2}G(\xi)}$$
$$= \alpha\log(\frac{\Lambda}{\mu})\tilde{J}_x(\alpha) \quad (36)$$

where

$$\tilde{J}_x(\alpha) = \int_0^\infty d\xi \frac{\xi^3}{(1+\xi^4)^{3/2}} \frac{1}{1+\frac{\alpha N}{2}G(\xi_{\boldsymbol{q}})}. \quad (37)$$

This integral is well-defined and finite as

$$\frac{\xi^3}{(1+\xi^4)^{3/2}} \frac{1}{1+\frac{\alpha N}{2}G(\xi_{\boldsymbol{q}})}\Big|_{\xi\to\infty} \to \frac{1}{\xi^3}$$
$$\frac{\xi^3}{(1+\xi^4)^{3/2}} \frac{1}{1+\frac{\alpha N}{2}G(\xi_{\boldsymbol{q}})}\Big|_{\xi\to 0} \to \xi^4. \quad (38)$$

Also $\tilde{J}_x(\alpha)$ is always positive as $G(\xi) > 0$.

On the other hand, we can evaluate $\tilde{J}_y$ similarly in $\bar{A} \ll 1$ and obtain

$$\tilde{J}_y = \alpha\log(\frac{\Lambda}{\mu})\tilde{J}_y(\alpha), \quad (39)$$

in which

$$\tilde{J}_y(\alpha) = \int_0^\infty \frac{d\xi}{\xi} \frac{1-5\xi^4}{(1+\xi^4)^{5/2}} \frac{1}{1+\frac{\alpha N}{2}G(\xi)}. \quad (40)$$

The integral is finite and well-defined as

$$\frac{1-5\xi^4}{(1+\xi^4)^{5/2}} \frac{1}{1+\frac{\alpha N}{2}G(\xi)}\Big|_{\xi\to\infty} \propto \frac{1}{\xi^7}$$
$$\frac{1-5\xi^4}{(1+\xi^4)^{5/2}} \frac{1}{1+\frac{\alpha N}{2}G(\xi)}\Big|_{\xi\to 0} \propto \frac{1}{c_x}. \quad (41)$$

Hence there is no more divergence in the renormalization in the inverse mass than $\propto \log(\frac{\Lambda}{\mu})$ in equation(39).

IV. DETAILED CALCULATION OF MOMENTUM SHELL RG

In this supplemental method, we will perform the one-loop RG by the momentum-shell method. We start with the following bare action $\mathcal{S} = \mathcal{S}_\psi + \mathcal{S}_\phi + \mathcal{S}_{\psi,\phi}$ present in the main text,

$$\mathcal{S}_\psi = \int_{\boldsymbol{k},\omega} \psi^\dagger_{\boldsymbol{k},\omega}(-i\omega + vk_x\sigma^x + Ak_y^2\sigma^y)\psi_{\boldsymbol{k},\omega},$$
$$\mathcal{S}_\phi = \int_{\boldsymbol{k},\omega} \frac{1}{2}\Big(\sqrt{\eta q_x^2 + \frac{q_y^2}{\eta}} + \tilde{\kappa} q_x^2\Big)|\phi(\boldsymbol{q},\omega)|^2,$$
$$\mathcal{S}_{\psi,\phi} = \int_{\boldsymbol{k},\omega}\int_{\boldsymbol{q},\Omega} ie\ \phi(\boldsymbol{q},\omega)\psi^\dagger_{\boldsymbol{k}+\boldsymbol{q}/2,\Omega+\omega}\psi_{\boldsymbol{k}-\boldsymbol{q}/2,\Omega}, \quad (42)$$

where η is introduced to facilitate the engineering dimensional analysis of the anisotropic scaling along x and y in the bare boson propagator. The physical value of η, as mentioned in the main text, is 1 because the (bare) Coulomb interaction is isotropic. On the other hand, we have introduced κ into the bare action to allow the possible anomalous screening term $\sim \sqrt{|q_x|}$, via the anomalous scaling dimension appearing in $\sim q_x^2$ term, under the RG steps.

We start with the dimensional analysis. First of all, we set the engineering dimensions $[x] = -z_1, [y] = -1$ and $[\tau] = -z_2$. Then it is straightforward to show

$$[v] = z - z_1,\ [A] = z - 2,\ [e^2] = z - \frac{1+z_2}{2},\\ [\eta] = 1 - z_1,\ [\Lambda] = 1,\ [\tilde{\kappa}] = \frac{1-3z_1}{2} \quad (43)$$

where Λ is the momentum cutoff in the momentum q_y. Here the fields have the following dimensions.

$$[\phi] = \frac{1}{2}\Big(z + \frac{1+z_1}{2}\Big),\ [\psi] = \frac{1+z_1}{2} \quad (44)$$

On the other hand, in terms of physical unit, we have

$$[v] = \frac{L}{T},\ [A] = \frac{L^2}{T},\ [e^2] = \frac{L}{T},\\ [\eta] = 1,\ [\Lambda] = \frac{1}{L},\ [\kappa] = L \quad (45)$$

The field operators have the following scaling dimensions.

$$[\phi] = \frac{1}{\sqrt{LT}},\ [\psi] = \frac{1}{\sqrt{L}} \quad (46)$$

From the above dimensional analysis we have the following dimensionless constants, which will be used to parametrize the renormalization flow

$$\alpha = \frac{e^2\sqrt{\eta}}{2\pi^2 v},\ \bar{A} = \frac{A\eta\Lambda}{v},\ \gamma = \frac{\tilde{\kappa}A^2\Lambda^3\sqrt{\eta}}{v}. \quad (47)$$

Before proceeding to the details of the RG calculation, we first note that η and e are protected under the RG process due to the non-analytic structure of the propagator of the boson and the gauge invariance. Hence, the only non-trivial corrections are the renormalizations of $\{v, A\}$ in the fermion propagator and κ in the boson propagator. The renormalization can be deduced from the self-energies of the fermion and the boson, which we calculate below.

We start with the fermion self-energy.

$$-\Sigma_f(\boldsymbol{k}, \omega) = v k_x \sigma^x I_1 + A k_y^2 \sigma^y I_2, \quad (48)$$

such that

$$I_1 = \frac{e^2}{2}\int_{\boldsymbol{q}} \frac{1}{\sqrt{\eta q_x^2 + \frac{q_y^2}{\eta} + \tilde{\kappa}q_x^2}} \frac{A q_y^4}{\Big[v^2 q_x^2 + A^2 q_y^4\Big]^{3/2}},\\ I_2 = \frac{e^2}{2}\int_{\boldsymbol{q}} \frac{1}{\sqrt{\eta q_x^2 + \frac{q_y^2}{\eta} + \tilde{\kappa}q_x^2}} \frac{v^2 q_x^2(v^2q_x^2 - 5A^2q_y^4)}{\Big[v^2 q_x^2 + A^2 q_y^4\Big]^{5/2}}, \quad (49)$$

where the integrals should be properly regularized as following.

$$\int_{\boldsymbol{q}} = \frac{1}{4\pi^2}\Big(\int_\mu^\Lambda dq_y + \int_{-\Lambda}^{-\mu} dq_y\Big)\int_{-\infty}^{\infty} dq_x,\\ = \frac{1}{\pi^2}\int_\mu^\Lambda dq_y \int_0^\infty dq_x, \quad (50)$$

as the integrands of equation(49) are even under $q_i \to -q_i, i = x, y$. Here $\mu = \Lambda e^{-l} \approx \Lambda(1-l)$ with $l \ll 1$ is assumed by following the standard strategy of the momentum-shell calculations.

With the assumption $\bar{A} \ll 1$ which will be justified self-consistently in the end of the calculation, we can evalute the integrals straightforwardly as following.

$$I_1 = \alpha l F_1[\gamma],\\ I_2 = \alpha l F_2[\gamma], \quad (51)$$

where

$$F_1[\gamma] = \int_0^\infty dx \frac{1}{1+\gamma x^2}\frac{1}{(x^2+1)^{3/2}},\\ F_2[\gamma] = \int_0^\infty dx \frac{1}{1+\gamma x^2}\frac{x^2(x^2-5)}{(x^2+1)^{5/2}}. \quad (52)$$

We remark that in $\gamma \to 0$ limit ($\tilde{\kappa} \to 0$), the inverse mass correction F_2 diverges logarithmically in $\sim \log\gamma$. This indicates instability of the non-interacting fermion ground state with the bare Coulomb interaction without operator insertions.

We now compute the polarization by the momentum-shell integral method

$$-\Pi(\boldsymbol{p}) = p_x^2 I_b, \quad (53)$$

in which

$$I_b = \frac{e^2}{4\pi^2}\int_\mu^\Lambda dk_y \int_0^\infty dk_x \frac{v^2A^2k_y^4}{[v^2k_x^2 + A^2k_y^4]^{5/2}},\\ = \frac{1}{3}\frac{\alpha}{A^2\Lambda^3\sqrt{\eta}/v^2} l. \quad (54)$$

Now we have the renormalized action $\mathcal{S}' = \mathcal{S}'_\psi + \mathcal{S}'_\phi + \mathcal{S}'_{\phi,\psi}$obtained by integrating out the high-energy modes as the following. We start with the fermion part $\mathcal{S}'_\psi$ where

$$\mathcal{S}'_\psi = \int d^3x\ \bar{\psi}(\partial_0 + ie\phi)\psi\\ + \int d^3x\ \bar{\psi}(v(1+\alpha F_1[\gamma]l)\gamma_1\partial_x - A(1+\alpha F_2[\gamma]l)(\partial_2)^2)\psi. \quad (55)$$

On the other hand, the boson part $\mathcal{S}'_\phi$ is

$$\mathcal{S}'_\phi = \int d^3x\ \frac{1}{2}\phi(\sqrt{\eta q_x^2 + \frac{q_y^2}{\eta}} + \tilde{\kappa}(1+\beta l)q_x^2)\phi, \quad (56)$$

where

$$\beta = \frac{1}{3}\frac{\alpha}{\gamma}. \quad (57)$$

On the other hand, the vertex correction vanishes and thus $\mathcal{S}'_{\phi,\psi}$ is the same as $\mathcal{S}_{\phi,\psi}$ from equation(42).

From these, we can obtain the flow equations.

$$\begin{aligned}
\frac{d}{dl}\log v &= z - z_1 + J_1 \\
\frac{d}{dl}\log A &= z - 2 + J_2 \\
\frac{d}{dl}\log \eta &= 1 - z_1 \\
\frac{d}{dl}\log \tilde{\kappa} &= \frac{1-3z_1}{2} + \beta \\
\frac{d}{dl}\log e^2 &= z - \frac{1+z_1}{2}
\end{aligned} \tag{58}$$

We now derive the following renormalization flow equations

$$\begin{aligned}
\frac{d\alpha}{dl} &= -\alpha^2 F_1[\gamma], \\
\frac{d\gamma}{dl} &= \frac{\alpha}{3} + \gamma[-3 + 2\alpha(F_2[\gamma] - F_1[\gamma])], \\
\frac{d\beta}{dl} &= \beta(3-\beta) + \alpha\beta[F_1[\gamma] - 2F_2[\gamma]].
\end{aligned} \tag{59}$$

The fixed point of these equations is $(\alpha, \gamma, \beta) \to (0, 0, 3)$. We plot the numerical solutions also in Fig.2. This also implies that $(\alpha F_1[\gamma], \alpha F_2[\gamma]) \to (0, 0)$. With this fixed point, we now derive the fixed point action $\mathcal{S}^f = \mathcal{S}^f_\psi + \mathcal{S}^f_\phi + \mathcal{S}^f_{\phi,\psi}$

$$\begin{aligned}
\mathcal{S}^f_\psi &= \int_{\boldsymbol{k},\omega} \psi^\dagger_{\boldsymbol{k},\omega}(-i\omega + vk_x\sigma^x + Ak_y^2\sigma^y)\psi_{\boldsymbol{k},\omega}, \\
\mathcal{S}^f_\phi &= \int_{\boldsymbol{k},\omega} \frac{1}{2}\Big(|\boldsymbol{q}| + \kappa\sqrt{|q_x|}\Big)|\phi(\boldsymbol{q},\omega)|^2, \\
\mathcal{S}^f_{\psi,\phi} &= \int_{\boldsymbol{k},\omega}\int_{\boldsymbol{q},\Omega} ie\;\phi(\boldsymbol{q},\omega)\psi^\dagger_{\boldsymbol{k}+\boldsymbol{q}/2,\Omega+\omega}\psi_{\boldsymbol{k}-\boldsymbol{q}/2,\Omega},
\end{aligned} \tag{60}$$

where we have used $\eta = 1$ for physical limit and $(1+\beta l)\Big|_{\beta=3} q_x^2 \approx q_x^2 e^{3l} \to \sqrt{|q_x|}$ because e^{-2l} is the scaling factor of q_x.

Now we justify $\bar{A} \ll 1$ which has been used in the derivations of the fermion self-energy. We calculate

$$\frac{d}{dl}\log \bar{A} = -1 + J_2 - J_1 \tag{61}$$

and we can approximate $J_2 = J_1 \approx 0$ near the fixed point. Then we clearly have

$$\frac{d}{dl}\log \bar{A} = -1 \tag{62}$$

which dictates that $\bar{A} \to 0 \ll 1$ at the fixed point.

V. STABILITY OF NEW FIXED POINT

In this supplemental method, we diagnose the stability and the scaling properties near the new intermediate fixed point. To determine the stability of the fixed point and the behavior near the fixed point more carefully, we now take equation(60) as the bare action and perform the momentum shell renormalization analysis again. Taking equation(60) as the bare action, we have

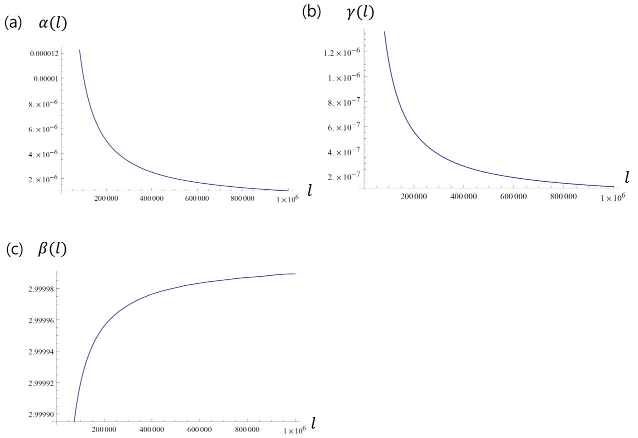

FIG. 2. Plot of RG flows. Here l represents the RG time. We have taken the initial conditions $\alpha[0] = \gamma[0] = 0.1$.

$$[\kappa] = \frac{1}{2} = \sqrt{\frac{1}{L}}. \tag{63}$$

Within the theory equation(60), we can introduce a dimensionless number

$$\bar{\kappa} = \kappa\sqrt{\frac{A\eta}{v}} \tag{64}$$

which will determine the renormalization flow near the fixed point equation(60). Due to the non-analyticity and the gauge invariance, $\{e, \eta, \kappa\}$ are protected under the renormalization process. Then the only non-trivial renormalizations occur in $\{v, A\}$, coming from the fermion self-energy. We again evaluate the fermion self-energy

$$-\Sigma_f(\boldsymbol{k},\omega) = vk_x\sigma^x I_1' + Ak_y^2\sigma^y I_2' \tag{65}$$

in which

$$\begin{aligned}
I_1' &= \frac{e^2}{2}\int_{\boldsymbol{q}} \frac{1}{\sqrt{\eta q_x^2 + \frac{q_y^2}{\eta}} + \kappa\sqrt{|q_x|}} \frac{A^2 q_y^4}{\Big[v^2q_x^2 + A^2q_y^4\Big]^{3/2}} \\
I_2' &= \frac{e^2}{2}\int_{\boldsymbol{q}} \frac{1}{\sqrt{\eta q_x^2 + \frac{q_y^2}{\eta}} + \kappa\sqrt{|q_x|}} \frac{v^2q_x^2(v^2q_x^2 - 5A^2q_y^4)}{\Big[v^2q_x^2 + A^2q_y^4\Big]^{5/2}}
\end{aligned} \tag{66}$$

where the momentum integral $\int_{\boldsymbol{q}}$ is regularized as in equation(50).

After the straightforward algebra with $\bar{A} \ll 1$ near the fixed point, we find

$$I_1' = \alpha l f_1[\bar{\kappa}]$$
$$I_2' = \alpha l f_2[\bar{\kappa}] \quad (67)$$

where

$$f_1[\bar{\kappa}] = \int_0^\infty dx \frac{1}{1+\bar{\kappa}\sqrt{x}} \frac{1}{(x^2+1)^{3/2}}$$
$$f_2[\bar{\kappa}] = \int_0^\infty dx \frac{1}{1+\bar{\kappa}\sqrt{x}} \frac{x^2(x^2-5)}{(x^2+1)^{5/2}}. \quad (68)$$

From these, we can derive the flow equations

$$\frac{d}{dl}\log v = z - z_1 + \alpha f_1[\bar{\kappa}]$$
$$\frac{d}{dl}\log A = z - 2 + \alpha f_2[\bar{\kappa}]$$
$$\frac{d}{dl}\log \eta = 1 - z_1$$
$$\frac{d}{dl}\log \kappa = \frac{1}{2}$$
$$\frac{d}{dl}\log e^2 = z - \frac{1+z_1}{2} \quad (69)$$

which implies

$$\frac{d}{dl}\alpha = -\alpha^2 f_1[\bar{\kappa}]$$
$$\frac{d}{dl}\bar{\kappa} = \frac{\alpha}{2}\bar{\kappa}[f_2[\bar{\kappa}] - f_1[\bar{\kappa}]]. \quad (70)$$

The attractive fixed point of these flow equations is $(\alpha, \bar{\kappa}) \to (0, \bar{\kappa}^* \approx 0.35)$ such that $f_2[\bar{\kappa}^*] = f_1[\bar{\kappa}^*]$. The numerical solution can be found in Fig.3. Hence we find that the velocity and inverse mass at the energy scale μ are

$$v(\mu) \approx v(1 + \alpha C_f \log(\frac{\Lambda}{\mu}))$$
$$A(\mu) \approx A(1 + \alpha C_f \log(\frac{\Lambda}{\mu})) \quad (71)$$

where $\mathcal{C}_f = f_1[\bar{\kappa}^*] \approx 0.8$. This logarithmic divergence as $\mu \to 0$ is reminiscent of graphene case.

VI. DETAILED CALCULATION OF SCREENING CHARGE

In this supplemental method, we show the detailed calculation of the screening charge dictated by equation (18) in the main text and equation (20) in the main text when a single impurity charge Z is introduced at $\boldsymbol{r} = 0$. We calculate the free and interacting cases at the level of the linear response theory. The details of the calculation here will closely follow Biswas, Son, and Sachdev[8].

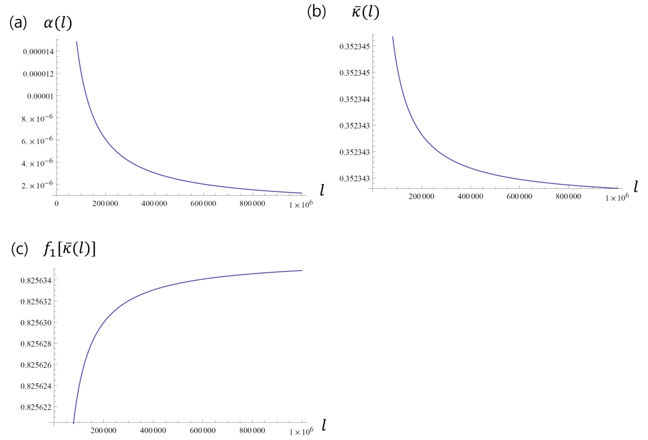

FIG. 3. Plot of RG flows. Here l represents the RG time. We have taken the initial conditions $\alpha[0] = \bar{\kappa}[0] = 0.5$.

2. *Free Theory*

We first compute the screening charge of the non-interacting theory from equation (7) in the main text. The screening charge is given by

$$\rho^f_{\text{ind}}(\boldsymbol{q}) = Z D_0(\boldsymbol{q}) \Pi_0(\boldsymbol{q}) \quad (72)$$

in which $D_0(\boldsymbol{q}) = \frac{1}{|\boldsymbol{q}|}$ and $\Pi_0(\boldsymbol{q})$ is the one-loop polarization. The real space configuration of the screening charge, we perform the Fourier transformation

$$\rho^f_{\text{ind}}(\boldsymbol{r}) = \int \frac{d^2q}{4\pi^2} e^{i\boldsymbol{q}\cdot\boldsymbol{r}} \rho^f_{\text{ind}}(\boldsymbol{q}) \quad (73)$$

We will be mainly interested in the directional behavior of the scaling in the integrated screening charges $Q_x(x)$ and $Q_y(y)$ along $\hat{x}$ and $\hat{y}$, which are obtained from

$$Q_x(x) = \int_{-\infty}^{\infty} dy \ \rho^f_{\text{ind}}(\boldsymbol{r})$$
$$Q_y(y) = \int_{-\infty}^{\infty} dx \ \rho^f_{\text{ind}}(\boldsymbol{r}) \quad (74)$$

We now evaluate these screening charges. We start with

$$Q_x(x) = \int_{-\infty}^{\infty} dy \ \rho^f_{\text{ind}}(\boldsymbol{r})$$
$$= \int \frac{dq_x}{2\pi} \rho^f_{\text{ind}}(q_x, 0) e^{iq_x x}$$
$$= Z \int \frac{dq_x}{2\pi} D_0(q_x, 0) \Pi_0(q_x, 0) e^{iq_x x} \quad (75)$$

Now from

$$\int_0^x Q_x(x')dx' \approx \rho^f_{\rm ind}(q_x = \frac{1}{|x|}) = ZD_0(q_x,0)\Pi_0(q_x,0)\Big|_{q_x=\frac{1}{|x|}} = -Z\frac{e^2}{4\pi^2}\frac{c_x}{\sqrt{vA}}\frac{1}{\sqrt{|q_x|}}\Big|_{q_x=\frac{1}{|x|}}, \quad (76)$$

we can take the derivative with respect to x on the both sides. After the straightforward calculation, we have

$$Q_x(x) = -\frac{Z\alpha c_x}{8\pi^2}\sqrt{\frac{v}{A}}\frac{1}{\sqrt{|x|}} \quad (77)$$

Similarly, we have the following expression for the other direction

$$Q_y(y) = Z\frac{\partial}{\partial y}\Big(D_0(0,q_y)\Pi_0(0,q_y)\Big)\Big|_{q_y=\frac{1}{|y|}}. \quad (78)$$

By plugging

$$D_0(0,q_y)\Pi_0(0,q_y) = \frac{e^2c_y}{4\pi^2 v}, \quad (79)$$

we find that

$$Q_y(y) \propto \delta(y). \quad (80)$$

Hence we see that the free theory has completely different scaling behaviors of the integrated screening charge along $\hat{x}$ and $\hat{y}$ directions, i.e.,

$$Q_x(x) \propto -\frac{Z}{\sqrt{x}},\ Q_y(y) \propto -Z\delta(y). \quad (81)$$

whose sign is the opposite of the impurity charge Z.

3. Interacting Theory

Now we use the non-trivial anisotropic fixed point equation (18) in the main text obtained from the interactions between the fermion and the boson. We will use the renormalized boson propagator dictated from equation (18) in the main text and the scaling behavior equation (20) in the main text to deduce the asymptotic behaviors of the integrated screening charges.

$$\rho^f_{\rm ind}(\boldsymbol{q}) \approx ZD_{\rm rem}(\boldsymbol{q})\Pi_0(\boldsymbol{q}), \qquad D_{\rm rem}(\boldsymbol{q})^{-1} = |\boldsymbol{q}| + \kappa\sqrt{|q_x|}. \quad (82)$$

From this, we now calculate the integrated screening charge $Q_x(x)$. To calculate it, we need

$$\Pi_0(q_x,0) = -\frac{e^2c_x}{4\pi^2 v(q_x)}\sqrt{\frac{v(q_x)}{A(q_x)}}\sqrt{|q_x|} \quad (83)$$

in which $\{v(q), A(q)\}$ are the running parameters at the scale q. From equation(71), we have

$$\Pi_0(q_x,0) = -\frac{e^2c_x}{4\pi^2 v(q_x)}\sqrt{\frac{v_0}{A_0}}\sqrt{|q_x|}, \quad (84)$$

where $\{v_0, A_0\}$ are the bare parameters. Hence we find

$$\rho_{\rm ind}(q_x) = -Z\frac{e^2c_x}{4\pi^2\kappa v(q_x)}\sqrt{\frac{v_0}{A_0}}, \quad (85)$$

where we have $v(q_x) = v_0(1+\alpha C_f\log(\frac{1}{r_0 q_x}))$ with the $r_0^{-1} \sim E_0$, the bandwidth of the bare action equation (6) in the main text. From the relationship

$$\int_0^x Q_x(x')dx' \approx \rho_{\rm ind}(q_x = \frac{1}{|x|}), \quad (86)$$

we find

$$Q_x(x) \approx \frac{Z(\alpha C_f)^2 c_x}{2f_1[\bar{\kappa}^*]}\sqrt{\frac{v_0}{A_0}}\frac{1}{|x|(1+\alpha C_f\log(|x|/r_0))^2} \propto \frac{Z}{|x|(1+\alpha C_f\log(|x|/r_0))^2}, \quad (87)$$

whose sign is the same as the impurity charge Z.

From

$$\Pi(0,q_y) = -\frac{e^2c_y}{4\pi^2 v(q_y)}|q_y|, \quad (88)$$

we find

$$\rho_{\rm ind}(0,q_y) = -\frac{e^2Zc_y}{4\pi^2 v(q_y)}. \quad (89)$$

Thus we see that

$$Q_y(y) \approx \frac{(\alpha C_f)^2 Zc_y}{2f_1[\bar{\kappa}^*]}\frac{1}{|y|(1+\alpha C_f\log(|y|/r_0))^2} \propto \frac{Z}{|y|(1+\alpha C_f\log(|y|/r_0))^2} \quad (90)$$

whose sign is the same as the impurity charge Z.

VII. DETAILED CALCULATION OF OPTICAL CONDUCTIVITY

In this supplemental method, we compute the optical conductivity $\sigma_{jj}(\Omega), j = x, y$ at the level of the linear response theory. The expression for the optical conductivity is given by

$$\sigma_{jj}(\Omega) = \frac{1}{\Omega}{\rm Im}\Big[\Pi^R_{jj}(\Omega+i\delta)\Big],\ j = x, y \quad (91)$$

in which $\Pi^R_{ii}(\Omega+i\delta) \equiv \Pi_{ii}(i\Omega \to \Omega+i\delta)$ where the latter is obtained from the imaginary-time formalism. Hence

we need to compute

$$\Pi_{jj}(i\Omega) = \int_{\vec{k},\omega} \mathrm{Tr}\Big[M_j g_f(\vec{k},\omega+\Omega) M_j g_f(\vec{k},\Omega)\Big], \\ = \sum_{\alpha,\beta=\pm1} \int_{\vec{k}} \mathrm{Tr}\Big(M_j P_{\vec{k},\alpha} M_j P_{\vec{k},\beta}\Big) \frac{n_F(\alpha)-n_F(\beta)}{-i\Omega+\alpha E_{\vec{k}}-\beta E_{\vec{k}}} \tag{92}$$

in which M_j is the appropriate vertex along the j-direction and the factor $P_{\vec{k},\alpha} = \frac{1}{2}\Big(1+\alpha\frac{H_{\vec{k}}}{E_{\vec{k}}}\Big), \alpha = \pm1$ comes from the Green's function $g_f(\vec{k},\omega)$. By performing summation over $\{\alpha,\beta\}$, it is straightforward to find

$$\Pi_{jj}(i\Omega) = \int_{\vec{k}} \mathrm{Tr}\Big(M_j P_{\vec{k},+1} M_j P_{\vec{k},-1}\Big) \times \Big(\frac{-1}{-i\Omega+2E_{\boldsymbol{k}}} + \frac{1}{-i\Omega+2E_{\boldsymbol{k}}}\Big) \tag{93}$$

Here we perform the Wick rotation $i\Omega \to \Omega + i\delta$.

$$\Pi^R_{jj}(\Omega) = \int_{\vec{k}} \mathrm{Tr}\Big(M_j P_{\vec{k},+1} M_j P_{\vec{k},-1}\Big) \times \Big(\frac{-1}{\Omega+i\delta-2E_{\boldsymbol{k}}} - \frac{1}{\Omega+i\delta+2E_{\boldsymbol{k}}}\Big) \tag{94}$$

Now it is straightforward to take the imaginary part from this expression. With the assumption $\Omega > 0$, we have

$$\mathrm{Im}\Big[\Pi^R_{jj}(\Omega)\Big] = \pi \int_{\vec{k}} \mathrm{Tr}\Big(M_j P_{\vec{k},+1} M_j P_{\vec{k},-1}\Big)\delta(\Omega-2E_{\boldsymbol{k}}), \tag{95}$$

which can be evaluated by performing elementary integrals over $\boldsymbol{k}$. By combining with equation(91), we finally obtain

$$\sigma_{jj}(\Omega) = \frac{\pi}{\Omega} \int_{\vec{k}} \mathrm{Tr}\Big(M_j P_{\vec{k},+1} M_j P_{\vec{k},-1}\Big)\delta(\Omega-2E_{\boldsymbol{k}}). \tag{96}$$

Now we evaluate this for $M_x = ve\sigma^x$ and $M_y = Aek_y\sigma^y$ to calculate $\sigma_{xx}(\Omega)$ and $\sigma_{yy}(\Omega)$ for this anisotropic semimetal.

Plugging $M_x = ve\sigma^x$ into equation(96), we have

$$\sigma_{xx}(\Omega) = \frac{e^2v^2}{8\pi\Omega} \int dk_x dk_y \mathrm{Tr}\Big(\sigma_x P_{\vec{k},+1} \sigma_x P_{\vec{k},-1}\Big) \times \delta(E_{\boldsymbol{k}} - \frac{\Omega}{2}) \tag{97}$$

By the change of variables $\zeta_x = vk_x$ and $\zeta_y = Ak_y^2$ and then performing the trace, we have

$$\sigma_{xx}(\Omega) = \frac{e^2 v}{16\pi\sqrt{A}\Omega} \int d\zeta_x d\zeta_y \delta(\sqrt{\zeta_x^2+\zeta_y^4} - \frac{\Omega}{2}) \times \Big(1 - \frac{\zeta_x^2-\zeta_y^4}{\zeta_x^2+\zeta_y^4}\Big) \tag{98}$$

Performing the change of the variables $\zeta_x = \Omega x$ and $\zeta_y = \sqrt{\Omega} y$, we find

$$\sigma_{xx}(\Omega) = \frac{e^2 v}{16\pi\sqrt{A}} \frac{I_{xx}}{\sqrt{\Omega}} \tag{99}$$

with the constant I_{xx} defined as following

$$I_{xx} = \int dxdy\ \delta(\sqrt{x^2+y^4} - \frac{1}{2})\Big(1 + \frac{y^4-x^2}{y^4+x^2}\Big), \\ \approx 2.47 \tag{100}$$

We can plug $M_y = Aek_y\sigma^y$ to calculate $\sigma_{yy}(\Omega)$. After the straightforward calculation, we find

$$\sigma_{yy}(\Omega) = \frac{e^2\sqrt{A}}{4\pi v} I_{yy}\sqrt{\Omega}, \tag{101}$$

where I_{yy} is a constant

$$I_{yy} = \int dxdy\ \delta(\sqrt{x^2+y^4} - \frac{1}{2})\Big(1 + \frac{x^2-y^4}{y^4+x^2}\Big), \\ \approx 4.94 \tag{102}$$

In summary, we found anisotropic scalings in the optical conductivities along $\hat{x}$ and $\hat{y}$ directions

$$\sigma_{xx}(\Omega) \propto 1/\sqrt{\Omega},\ \ \sigma_{yy}(\Omega) \propto \sqrt{\Omega}, \tag{103}$$

VIII. INDEPENDENCE OF CUTOFF SCHEME AT QCP

In this supplementary material, we show clearly that the RG flow equations near the QCP in V, i.e., Eq(8) in main text and Eq(69) of V, are cutoff scheme independent. To show this, we first note that the only nontrivial renormalization to the bare action (60) is from the fermion self-energy, and thus we only need to show the independence of the cutoff scheme in the renormalization of the velocity v and the mass A, which can be calculated from

$$-D_\Lambda \Sigma_f(\boldsymbol{k},\omega) = -\Lambda \frac{d}{d\Lambda}\Sigma_f(\boldsymbol{k},\omega). \tag{104}$$

We introduce a soft cutoff function $J(\frac{q_y}{\Lambda}) = J(-\frac{q_y}{\Lambda})$ which is a smooth function (which is monotonically decreasing for $q_y > 0$) satisfying

$$J(0) = 1, \quad J(\infty) = 0. \tag{105}$$

Then the fermion self-energy can be written as

$$-\Sigma_f(\boldsymbol{k},\omega) = \frac{e^2}{2} \int_{\boldsymbol{q}} \frac{H(\boldsymbol{q}+\boldsymbol{k})}{E(\boldsymbol{q}+\boldsymbol{k})} g_b(\boldsymbol{q}) J(\frac{q_y+k_y}{\Lambda}) J(\frac{q_y}{\Lambda}). \tag{106}$$

We will explicitly demonstrate that the renormalizations to the velocity v and mass A are independent of the detailed form of the function $J(\cdot)$, i.e., the flow equations near the QCP (see V),

$$\frac{d}{dl}\log v = z - z_1 + \alpha f_1[\bar{\kappa}]$$
$$\frac{d}{dl}\log A = z - 2 + \alpha f_2[\bar{\kappa}] \tag{107}$$

with

$$f_1[\bar{\kappa}] = \int_0^\infty dx \frac{1}{1+\bar{\kappa}\sqrt{x}} \frac{1}{(x^2+1)^{3/2}}$$
$$f_2[\bar{\kappa}] = \int_0^\infty dx \frac{1}{1+\bar{\kappa}\sqrt{x}} \frac{x^2(x^2-5)}{(x^2+1)^{5/2}}, \tag{108}$$

are independent of the precise form of the cutoff function $J(\cdot)$. Note that the flow equations in V are originally obtained by using the hard cutoff function.

A. Correction to the velocity v: we first calculate the correction to the velocity. We only need to calculate

$$-\Sigma_f(\boldsymbol{k},\omega) = \frac{e^2}{2}\int_{\boldsymbol{q}} \frac{H(\boldsymbol{q}+\boldsymbol{k})}{E(\boldsymbol{q}+\boldsymbol{k})} g_b(\boldsymbol{q}) J^2(\frac{q_y}{\Lambda}), \tag{109}$$

with $\boldsymbol{k}=(k_x,0)$. Expanding in $\frac{H(\boldsymbol{q}+\boldsymbol{k})}{E(\boldsymbol{q}+\boldsymbol{k})}$ to $O(k_x)$, we find

$$-\Sigma_f(\boldsymbol{k},\omega) = v k_x \sigma^x \times I_x, \tag{110}$$

where

$$I_x = \frac{e^2}{2\pi^2}\int_0^\infty dq_x \int_0^\infty dq_y \Big(\frac{A^2 q_y^4}{[v^2q_x^2 + A^2 q_y^4]^{3/2}} \times \frac{1}{q_y+\kappa\sqrt{q_x}} J^2(\frac{q_y}{\Lambda})\Big). \tag{111}$$

We now perform the derivative D_Λ on the self-energy to find

$$-D_\Lambda[\Sigma_f(\boldsymbol{k},\omega)] = v k_x \sigma^x \times D_\Lambda[I_x]. \tag{112}$$

Hence we calculate

$$D_\Lambda[I_x] = \frac{e^2}{2\pi^2}\int_0^\infty dq_x \int_0^\infty dq_y \Big(\frac{A^2 q_y^4}{[v^2q_x^2 + A^2 q_y^4]^{3/2}} \times \frac{1}{q_y+\kappa\sqrt{q_x}} D_\Lambda[J^2(\frac{q_y}{\Lambda})]\Big). \tag{113}$$

To evaluate the integral, we first perform the change of the variable $y=\frac{q_y}{\Lambda}$ to find

$$D_\Lambda[I_x] = -\frac{e^2}{2\pi^2}\Lambda\times\int_0^\infty dq_x \int_0^\infty dy \Big(\frac{A^2\Lambda^4 y^4}{[v^2q_x^2 + A^2\Lambda^4 y^4]^{3/2}} \times \frac{1}{\Lambda y+\kappa\sqrt{q_x}} \times y\frac{d}{dy}[J^2(y)]\Big). \tag{114}$$

Next we scale out $q_x \to q_x y$ to find

$$D_\Lambda[I_x] = -\frac{e^2}{2\pi^2}\Lambda\times \int_0^\infty dq_x \Big(\frac{A^2\Lambda^4}{[v^2q_x^2 + A^2\Lambda^4]^{3/2}} \frac{1}{\Lambda+\kappa\sqrt{q_x}}\Big) \int_0^\infty dy \frac{d}{dy}[J^2(y)], \tag{115}$$

where the integral over y can be performed analyticall to find

$$D_\Lambda[I_x] = \frac{e^2}{2\pi^2}\Lambda\times \int_0^\infty dq_x \Big(\frac{A^2\Lambda^4}{[v^2q_x^2 + A^2\Lambda^4]^{3/2}} \frac{1}{\Lambda+\kappa\sqrt{q_x}}\Big), \tag{116}$$

which is nothing but the momentum-shell integral for the renormalization of the velocity v obtained in (66) of V, i.e., I_1' in Eq.(66). Notice that the expression is manifestly independent of the precise form of $J(\cdot)$. After successive change of variables as in V, we can show that the same integral $f_1[\bar{\kappa}]$ in Eq.(107) appears in the renormalization of the velocity v.

B. Correction to the mass A: we next calculate the correction to the mass A. After the straightforward series expansion of (109) for $\boldsymbol{k}=(0,k_y)$ to $O(k_y^2)$ and then taking D_Λ, we find

$$-D_\Lambda[\Sigma_f(\boldsymbol{k},\omega)] = A k_y^2 \sigma^y (K_1+K_2+K_3), \tag{117}$$

where $\{K_1,K_2,K_3\}$ are the following integrals to be evaluated.

$$K_1 = \frac{e^2}{2A}\int_{\boldsymbol{q}} g_b(\boldsymbol{q})\Big(\frac{1}{\Lambda^3}f(\boldsymbol{q})\times \Big(\Lambda J(\frac{q_y}{\Lambda})J''(\frac{q_y}{\Lambda}) + \frac{q_y}{2}J'(\frac{q_y}{\Lambda})J''(\frac{q_y}{\Lambda}) + \frac{q_y}{2}J(\frac{q_y}{\Lambda})J'''(\frac{q_y}{\Lambda})\Big)\Big), \tag{118}$$

in which $f(\boldsymbol{q}) = \frac{A q_y^2}{\sqrt{v^2q_x^2+A^2q_y^4}}$ and we have used the notation $f'(x)=\frac{d}{dx}f(x)$ and similarly for $f''(x), f'''(x)$. For K_2, we have

$$K_2 = \frac{e^2}{2A}\int_{\boldsymbol{q}} g_b(\boldsymbol{q})\Big(\frac{1}{\Lambda^3}f'(\boldsymbol{q})\times \Big(\Lambda^2 J(\frac{q_y}{\Lambda})J'(\frac{q_y}{\Lambda}) + q_y\Lambda[J'(\frac{q_y}{\Lambda})]^2 + q_y\Lambda J(\frac{q_y}{\Lambda})J''(\frac{q_y}{\Lambda})\Big)\Big), \tag{119}$$

and, for K_3, we have

$$K_3 = \frac{e^2}{2A}\int_{\boldsymbol{q}} \frac{g_b(\boldsymbol{q})}{\Lambda} f''(\boldsymbol{q}) q_y J(\frac{q_y}{\Lambda})J'(\frac{q_y}{\Lambda}). \tag{120}$$

Performing the change of the variable $q_y=\Lambda y$ and then scaling $q_x\to q_x y$, we can show that

$$K_1 \propto \int_0^\infty dy \frac{d}{dy}[y^2 J(y)J''(y)] = y^2 J(y)J''(y)\Big|_0^\infty = 0,$$
$$K_2 \propto \int_0^\infty dy \frac{d}{dy}[y J(y)J'(y)] = y^2 J(y)J'(y)\Big|_0^\infty = 0. \tag{121}$$

On the other hand, we find

$$K_3 = -\frac{e^2}{2\pi^2}\Lambda \int_0^\infty dq_x \frac{1}{\kappa\sqrt{q_x}+\Lambda}\frac{v^2q_x^2[v^2q_x^2-5A^2\Lambda^4]}{[v^2q_x^2+A^2\Lambda^4]^{5/2}} \times \int_0^\infty dy \frac{d}{dy}[J^2(y)]. \quad (122)$$

By performing the integral over y explicitly, we find

$$K_3 = \frac{e^2}{2\pi^2}\Lambda \int_0^\infty dq_x \frac{1}{\kappa\sqrt{q_x}+\Lambda}\frac{v^2q_x^2[v^2q_x^2-5A^2\Lambda^4]}{[v^2q_x^2+A^2\Lambda^4]^{5/2}}, \quad (123)$$

which is nothing but the momentum-shell integral for the renormalization of the mass A obtained in (66) of V, i.e., I_2' in Eq.(66). Notice that the expression is manifestly independent of the precise form of $J(\cdot)$. After successive change of variables as in V, we can show that the same integral $f_2[\bar{\kappa}]$ in Eq.(107) appears in the renormalization of the mass A.

IX. WARD IDENTITY AND NON-RENORMALIZATION OF ELECTRIC CHARGE

Here, we discuss the Ward identity and its consequences in two dimensional systems with the Coulomb interaction, and we also discuss difference between our work and Isobe *et. al.*[9]. The effective action after redefinition $e\phi \to \phi$ is

$$\mathcal{S} = \int_{\boldsymbol{x},\tau} \psi^\dagger\big((\partial_\tau + i\phi) + \mathcal{H}(-i\nabla)\big)\psi + \int_{q,\omega} \frac{|q|}{2e^2}|\phi(q,\omega)|^2 + \mathcal{S}_{ins},$$

where $\mathcal{S}_{ins}$ represents an additional inserted operator. For the discussion of the Ward identity, the inserted operator part is not important but it becomes crucial to investigate the stability of the non-interacting fixed point. Note that the action has gauge-invariance under

$$\phi \to \phi + \partial_\tau\alpha(\tau), \quad \psi \to \psi e^{-i\alpha(\tau)}.$$

After incorporating quantum fluctuation, the effective action becomes

$$\mathcal{S} \to \int (1+\delta_\omega)\psi^\dagger\partial_\tau\psi + (1+\delta_v)\int i\phi\,\psi^\dagger\psi + \int \psi^\dagger\tilde{H}(-i\nabla)\psi + \int \frac{|q|}{2e^2}|\phi|^2 + \mathcal{S}_{ins},$$

Notice that non-analytic dependence of the $\frac{1}{e^2}|\boldsymbol{q}|$ term in $\boldsymbol{q}$ prohibits its renormalization under integrating out the high-energy modes. Here we did not explicitly write out the renormalized Hamiltonian $\tilde{H}(-i\nabla)$ as it is not important in the discussion of the Ward identity.

It is clear that the gauge invariance gives the Ward identity,

$$\delta_\omega = \delta_v.$$

Moreover the non-renormalizable e^2 gives that the correction terms are absorbed by redefining ψ, $\psi = \sqrt{Z_\psi}\psi_r$ with $Z_\psi^{-1} = 1+\delta_\omega$, and parameters in the Hamiltonian $\tilde{H}(-i\nabla)$ such as velocity and effective mass.

Therefore, in our calculation, the electric charge is always non-renormalizable but eZ_ψ receives correction even though it appears in higher loop calculation with the bare Coulomb interaction.

This is one main difference between work by Isobe *et. al.*[9] and ours. In their work, they claim electric charge (g in their notation) is renormalized but gZ (eZ_ψ in our notation) is not.

Another related difference between our work here and theirs[9] is their emphasis on the wavefunction renormalization $Z = (1+\frac{\partial\Sigma_f(\omega,\boldsymbol{k})}{\partial(i\omega)})^{-1}$. However, in the strong-coupling limit $\alpha N \to \infty$, it is not difficult to see that $\frac{\partial\Sigma_f(\omega,\boldsymbol{k})}{\partial(i\omega)}$ contains the log-squared divergence (see below for the detail), which originates from the poor screening of the Coulomb interaction at finite frequency with zero momentum. In this limit, the only effect of the Coulomb interaction is to rotate the phase of the fermions as well explained in Son[1] and thus should not be taken as physical. Such log-squared dependence is also present in Isobe *et.al.*[9] in the limit $\alpha N \to \infty$ and taken to calculate the fermion wavefunction renormalization Z. They interpret the wavefunction renormalization as single-particle residue. But it cannot be adiabatically connected to one of the strong coupling fixed point due to the additional divergence.

One can see another difference from insertion operators S_{ins}. As shown above, the perturbative RG with the bare Coulomb potential without the inserted operator gives log-squared divergence in the inverse effective mass correction. This log-squared correction indicates necessity of additional operators. In our calculation, we insert the operator

$$\mathcal{S}_{ins} = \frac{\tilde{\kappa}}{2}\int (\nabla\phi)^2,$$

which is natural in the Wilsonian RG, while they[9] insert an approximated polarization function even for weak coupling analysis,

$$\mathcal{S}'_{ins} = \int \frac{-1}{e^2}\Pi(q,\omega)|\phi(q,\omega)|^2.$$

We believe that the insertion of an approximated polarization function is questionable in the weak coupling limit since it is not controlled by the $\frac{1}{N_f}$ factor, which disappeared in their weak coupling analysis.

Note that the insertion of the approximated polarization which is contributed by the fermion modes from all energy scales is valid in strong coupling limit since it

is well controlled by the $\frac{1}{N_f}$ factor. In the current problem, the strong coupling limit is indeed controlled, and all physical quantities receives $\frac{1}{N_f}$ corrections which are well captured in Isobe et. al. and ours with qualitative difference from different approximation. However, as shown in the main text, the strong coupling fixed point becomes immediately unstable due to the anomalous dimension of the velocity, thus the validity of the large-N_f expansion is not guaranteed in the infrared limit.

We also believe that unusual cutoff (scale) dependence of physical quantities in their analysis (for example, $\log\alpha$ in RG equations) indicates their RG scheme is not conventional and it cannot be perturbatively obtained from the non-interacting theory. On contrary, in our analysis, all physical quantities receive logarithmic cutoff (scale) dependence as ones of quantum criticality described by renormalizable field theories.

Apart from the differences, we find the ground state and its excitation in the IR limit are marginally stable, so-called marginal Fermi liquid, which is also obtained by Isobe *et. al.*[9] in spite of different renormalization of the inverse mass.

A. Log-squared Divergence in Fermion Self-Energy

Here we present the log-squared divergence in the strong-coupling limit, $\alpha N_f \to \infty$, where $g_b^{-1}(\boldsymbol{q},\Omega) \to -\Pi(\boldsymbol{q},\Omega)$ by keeping the dependence in Ω.

Such log-squared divergence has been observed in the graphene case as discussed by Son[1]. The fermion self-energy in Son is given by

$$\Sigma(\boldsymbol{p},p_0) = \Sigma_0\gamma_0 p_0 + \Sigma_1 \boldsymbol{\gamma}\cdot\boldsymbol{p}, \tag{124}$$

where

$$\Sigma_0 = \frac{8}{N_f}\int \frac{d^2\boldsymbol{q}dq_0}{(2\pi)^3}\frac{q_0^2-\boldsymbol{q}^2}{(q^2)^{3/2}|\boldsymbol{q}|^2}, \tag{125}$$

with $q = |(q_0,\boldsymbol{q})|$, the size of the three-component vector $(q_0,\boldsymbol{q})$, which is not to be confused with the size of the spatial two-component vector $|\boldsymbol{q}| = |(q_x,q_y)|$. Naively, this expression is single-log divergent, but it is in fact log-squared divergent. The log-squared divergence is apparent in the limit $\frac{|\boldsymbol{q}|}{q_0} \to 0$ because Eq.(134) can be approximated

$$\begin{aligned}\Sigma_0 &\propto \int \frac{d^2\boldsymbol{q}dq_0}{(2\pi)^3}\frac{q_0^2}{(q_0^2)^{3/2}|\boldsymbol{q}|^2},\\ &\propto \int\frac{dq_0}{q_0}\times\int\frac{d^2\boldsymbol{q}}{|\boldsymbol{q}|^2},\end{aligned} \tag{126}$$

which has the two sources of the logarithmic divergence, one from the integral over q_0 and the other from the integral over $\boldsymbol{q}$.

We expect the similar log-squared divergence to be present if we keep the dependence of the polarization $\Pi(\boldsymbol{q},\Omega)$ in Ω. To exhibit this explicitly, we reconsider the fermion self-energy and the boson polarization in the strong-coupling limit $\alpha\to\infty$. Here we show the log-sqaured divergence in Σ_0 where

$$\Sigma_0 = -\frac{1}{i\omega}\mathrm{Tr}[\Sigma_f(\boldsymbol{k},\omega)]. \tag{127}$$

Here we work in the unit $v = A = 1$ for convenience and the clarity of the discussion. We start from the expression

$$\Sigma_0 \propto \int_{\boldsymbol{q},\Omega}\frac{\Omega^2-E_{\boldsymbol{q}}^2}{(\Omega^2+E_{\boldsymbol{q}})^2}\frac{1}{\Pi(\boldsymbol{q},\Omega)}, \tag{128}$$

where $E_{\boldsymbol{q}} = (q_x^2+q_y^4)^{1/2}$. In the limit $\Omega\gg E_{\boldsymbol{q}}$, we have

$$\begin{aligned}\Sigma_0 &\propto \int_{\boldsymbol{q},\Omega}\frac{1}{\Omega^2}\frac{1}{\Pi(\boldsymbol{q},\Omega)},\\ &\propto \int_\Omega\frac{1}{\Omega}\times\int_{\boldsymbol{q}}\frac{1}{a_x\frac{q_x^2}{\sqrt{|\Omega|}}+a_y q_y^2\sqrt{|\Omega|}},\end{aligned} \tag{129}$$

where $a_x\approx 0.66$ and $a_y\approx 0.75$ and we have used the following form of the polarization

$$\Pi(\boldsymbol{q},\Omega)\propto\frac{1}{\Omega}\Big[a_x\frac{q_x^2}{\sqrt{|\Omega|}}+a_y q_y^2\sqrt{|\Omega|}\Big], \tag{130}$$

which is asymptotically correct as far as $\Omega\gg E_{\boldsymbol{q}}$. Now in Eq.(138), we can perform the change of the variables $x = \frac{\sqrt{a_x}}{|\Omega|^{1/4}}q_x$ and $y = \sqrt{a_y}|\Omega|^{1/4}q_y$ to find

$$\Sigma_0\propto\int\frac{d\Omega}{\Omega}\times\int\frac{dxdy}{x^2+y^2}, \tag{131}$$

which clearly exhibits the log-sqaured divergence.

Now we show how we obtained the asymptotic expression of the boson self-energy Eq.(139). We start with the expression for the boson self-energy Eq.(5). By performing the following change of the variables $k_x = |\Omega|x$, $k_y = |\Omega|^{1/2}y$, $X = \frac{q_x}{|\Omega|}$ and $Y = \frac{q_y}{\sqrt{|\Omega|}}$ (remember that we took $v = A = 1$ for this subsection) and expanding Π to the lowest orders in X and Y, we find

$$\begin{aligned}\Pi(\boldsymbol{q},\Omega) &\approx \sqrt{|\Omega|}\Big[a_xX^2+a_yY^2\Big]\\ &= \frac{1}{\Omega}\Big[a_x\frac{q_x^2}{\sqrt{|\Omega|}}+a_y\sqrt{|\Omega|}q_y^2\Big].\end{aligned} \tag{132}$$

X. WARD IDENTITY AND NON-RENORMALIZATION OF ELECTRIC CHARGE

Here, we discuss the Ward identity and its consequences in two dimensional systems with the Coulomb interaction, and we also discuss difference between our

work and Isobe *et. al.*[9]. The effective action after redefinition $e\phi \to \phi$ is

$$\mathcal{S} = \int_{\boldsymbol{x},\tau} \psi^\dagger\big((\partial_\tau + i\phi) + \mathcal{H}(-i\nabla)\big)\psi + \int_{q,\omega} \frac{|q|}{2e^2}|\phi(q,\omega)|^2 + \mathcal{S}_{ins},$$

where $\mathcal{S}_{ins}$ represents an additional inserted operator. For the discussion of the Ward identity, the inserted operator part is not important but it becomes crucial to investigate the stability of the non-interacting fixed point. Note that the action has gauge-invariance under

$$\phi \to \phi + \partial_\tau\alpha(\tau), \quad \psi \to \psi e^{-i\alpha(\tau)}.$$

After incorporating quantum fluctuation, the effective action becomes

$$\mathcal{S} \to \int (1+\delta_\omega)\psi^\dagger\partial_\tau\psi + (1+\delta_v)\int i\phi\,\psi^\dagger\psi + \int \psi^\dagger\tilde{H}(-i\nabla)\psi + \int \frac{|q|}{2e^2}|\phi|^2 + \mathcal{S}_{ins},$$

Notice that non-analytic dependence of the $\frac{1}{e^2}|\boldsymbol{q}|$ term in $\boldsymbol{q}$ prohibits its renormalization under integrating out the high-energy modes. Here we did not explicitly write out the renormalized Hamiltonian $\tilde{H}(-i\nabla)$ as it is not important in the discussion of the Ward identity.

It is clear that the gauge invariance gives the Ward identity,

$$\delta_\omega = \delta_v.$$

Moreover the non-renormalizable e^2 gives that the correction terms are absorbed by redefining ψ, $\psi = \sqrt{Z_\psi}\psi_r$ with $Z_\psi^{-1} = 1 + \delta_\omega$, and parameters in the Hamiltonian $\tilde{H}(-i\nabla)$ such as velocity and effective mass.

Therefore, in our calculation, the electric charge is always non-renormalizable but eZ_ψ receives correction even though it appears in higher loop calculation with the bare Coulomb interaction.

This is one main difference between work by Isobe *et. al.*[9] and ours. In their work, they claim electric charge (g in their notation) is renormalized but gZ (eZ_ψ in our notation) is not.

Another related difference between our work here and theirs[9] is their emphasis on the wavefunction renormalization $Z = (1 + \frac{\partial\Sigma_f(\omega,\boldsymbol{k})}{\partial(i\omega)})^{-1}$. However, in the strong-coupling limit $\alpha N \to \infty$, it is not difficult to see that $\frac{\partial\Sigma_f(\omega,\boldsymbol{k})}{\partial(i\omega)}$ contains the log-squared divergence (see below for the detail), which originates from the poor screening of the Coulomb interaction at finite frequency with zero momentum. In this limit, the only effect of the Coulomb interaction is to rotate the phase of the fermions as well explained in Son[1] and thus should not be taken as physical. Such log-squared dependence is also present in Isobe *et.al.*[9] in the limit $\alpha N \to \infty$ and taken to calculate the fermion wavefunction renormalization Z. They interpret the wavefunction renormalization as single-particle residue. But it cannot be adiabatically connected to one of the strong coupling fixed point due to the additional divergence.

One can see another difference from insertion operators S_{ins}. As shown above, the perturbative RG with the bare Coulomb potential without the inserted operator gives log-squared divergence in the inverse effective mass correction. This log-squared correction indicates necessity of additional operators. In our calculation, we insert the operator

$$\mathcal{S}_{ins} = \frac{\tilde{\kappa}}{2}\int (\nabla\phi)^2,$$

which is natural in the Wilsonian RG, while they[9] insert an approximated polarization function even for weak coupling analysis,

$$\mathcal{S}'_{ins} = \int \frac{-1}{e^2}\Pi(q,\omega)|\phi(q,\omega)|^2.$$

We believe that the insertion of an approximated polarization function is questionable in the weak coupling limit since it is not controlled by the $\frac{1}{N_f}$ factor, which disappeared in their weak coupling analysis.

Note that the insertion of the approximated polarization which is contributed by the fermion modes from all energy scales is valid in strong coupling limit since it is well controlled by the $\frac{1}{N_f}$ factor. In the current problem, the strong coupling limit is indeed controlled, and all physical quantities receives $\frac{1}{N_f}$ corrections which are well captured in Isobe et. al. and ours with qualitative difference from different approximation. However, as shown in the main text, the strong coupling fixed point becomes immediately unstable due to the anomalous dimension of the velocity, thus the validity of the large-N_f expansion is not guaranteed in the infrared limit.

We also believe that unusual cutoff (scale) dependence of physical quantities in their analysis (for example, $\log\alpha$ in RG equations) indicates their RG scheme is not conventional and it cannot be perturbatively obtained from the non-interacting theory. On contrary, in our analysis, all physical quantities receive logarithmic cutoff (scale) dependence as ones of quantum criticality described by renormalizable field theories.

Apart from the differences, we find the ground state and its excitation in the IR limit are marginally stable, so-called marginal Fermi liquid, which is also obtained by Isobe *et. al.*[9] in spite of different renormalization of the inverse mass.

A. Log-squared Divergence in Fermion Self-Energy

Here we present the log-squared divergence in the strong-coupling limit, $\alpha N_f \to \infty$, where $g_b^{-1}(\boldsymbol{q},\Omega) \to -\Pi(\boldsymbol{q},\Omega)$ by keeping the dependence in Ω.

Such log-squared divergence has been observed in the graphene case as discussed by Son[1]. The fermion self-energy in Son is given by

$$\Sigma(\boldsymbol{p}, p_0) = \Sigma_0\gamma_0 p_0 + \Sigma_1 \boldsymbol{\gamma}\cdot\boldsymbol{p}, \tag{133}$$

where

$$\Sigma_0 = \frac{8}{N_f}\int \frac{d^2\boldsymbol{q}dq_0}{(2\pi)^3}\frac{q_0^2-\boldsymbol{q}^2}{(q^2)^{3/2}|\boldsymbol{q}|^2}, \tag{134}$$

with $q = |(q_0, \boldsymbol{q})|$, the size of the three-component vector $(q_0, \boldsymbol{q})$, which is not to be confused with the size of the spatial two-component vector $|\boldsymbol{q}| = |(q_x, q_y)|$. Naively, this expression is single-log divergent, but it is in fact log-squared divergent. The log-squared divergence is apparent in the limit $\frac{|\boldsymbol{q}|}{q_0} \to 0$ because Eq.(134) can be approximated

$$\begin{aligned}\Sigma_0 &\propto \int \frac{d^2\boldsymbol{q}dq_0}{(2\pi)^3}\frac{q_0^2}{(q_0^2)^{3/2}|\boldsymbol{q}|^2},\\ &\propto \int\frac{dq_0}{q_0}\times\int\frac{d^2\boldsymbol{q}}{|\boldsymbol{q}|^2},\end{aligned} \tag{135}$$

which has the two sources of the logarithmic divergence, one from the integral over q_0 and the other from the integral over $\boldsymbol{q}$.

We expect the similar log-squared divergence to be present if we keep the dependence of the polarization $\Pi(\boldsymbol{q}, \Omega)$ in Ω. To exhibit this explicitly, we reconsider the fermion self-energy and the boson polarization in the strong-coupling limit $\alpha \to \infty$. Here we show the log-sqaured divergence in Σ_0 where

$$\Sigma_0 = -\frac{1}{i\omega}\mathrm{Tr}[\Sigma_f(\boldsymbol{k}, \omega)]. \tag{136}$$

Here we work in the unit $v = A = 1$ for convenience and the clarity of the discussion. We start from the expression

$$\Sigma_0 \propto \int_{\boldsymbol{q},\Omega}\frac{\Omega^2 - E_{\boldsymbol{q}}^2}{(\Omega^2+E_{\boldsymbol{q}})^2}\frac{1}{\Pi(\boldsymbol{q},\Omega)}, \tag{137}$$

where $E_{\boldsymbol{q}} = (q_x^2 + q_y^4)^{1/2}$. In the limit $\Omega \gg E_{\boldsymbol{q}}$, we have

$$\begin{aligned}\Sigma_0 &\propto \int_{\boldsymbol{q},\Omega}\frac{1}{\Omega^2}\frac{1}{\Pi(\boldsymbol{q},\Omega)},\\ &\propto \int_\Omega \frac{1}{\Omega}\times\int_{\boldsymbol{q}}\frac{1}{a_x\frac{q_x^2}{\sqrt{|\Omega|}} + a_y q_y^2\sqrt{|\Omega|}},\end{aligned} \tag{138}$$

where $a_x \approx 0.66$ and $a_y \approx 0.75$ and we have used the following form of the polarization

$$\Pi(\boldsymbol{q},\Omega) \propto \frac{1}{\Omega}\Big[a_x\frac{q_x^2}{\sqrt{|\Omega|}} + a_y q_y^2\sqrt{|\Omega|}\Big], \tag{139}$$

which is asymptotically correct as far as $\Omega \gg E_{\boldsymbol{q}}$. Now in Eq.(138), we can perform the change of the variables $x = \frac{\sqrt{a_x}}{|\Omega|^{1/4}}q_x$ and $y = \sqrt{a_y}|\Omega|^{1/4}q_y$ to find

$$\Sigma_0 \propto \int\frac{d\Omega}{\Omega}\times\int\frac{dxdy}{x^2+y^2}, \tag{140}$$

which clearly exhibits the log-sqaured divergence.

Now we show how we obtained the asymptotic expression of the boson self-energy Eq.(139). We start with the expression for the boson self-energy Eq.(5). By performing the following change of the variables $k_x = |\Omega|x$, $k_y = |\Omega|^{1/2}y$, $X = \frac{q_x}{|\Omega|}$ and $Y = \frac{q_y}{\sqrt{|\Omega|}}$ (remember that we took $v = A = 1$ for this subsection) and expanding Π to the lowest orders in X and Y, we find

$$\begin{aligned}\Pi(\boldsymbol{q},\Omega) &\approx \sqrt{|\Omega|}\Big[a_x X^2 + a_y Y^2\Big]\\ &= \frac{1}{\Omega}\Big[a_x\frac{q_x^2}{\sqrt{|\Omega|}} + a_y\sqrt{|\Omega|}q_y^2\Big].\end{aligned} \tag{141}$$

[1] D. Son, Physical Review B **75**, 235423 (2007).
[2] J. González, F. Guinea, and M. Vozmediano, Physical Review B **59**, R2474 (1999).
[3] P. Hosur, S. Parameswaran, and A. Vishwanath, Physical review letters **108**, 046602 (2012).
[4] E.-G. Moon, C. Xu, Y. B. Kim, and L. Balents, Physical review letters **111**, 206401 (2013).
[5] S.-K. Jian and H. Yao, arXiv preprint arXiv:1503.07429 (2015).
[6] H.-H. Lai, arXiv preprint arXiv:1502.02695 (2015).
[7] B.-J. Yang, E.-G. Moon, H. Isobe, and N. Nagaosa, Nature Physics (2014).
[8] R. R. Biswas, S. Sachdev, and D. T. Son, Physical Review B **76**, 205122 (2007).
[9] Hiroki Isobe, Bohm-Jung Yang, Andrey Chubukov, Jörg Schmalian, and Naoto Nagaosa, arXiv:1508.03781 .
